# Bioreduction of 4′-Hydroxychalcone in Deep Eutectic Solvents: Optimization and Efficacy with Various Yeast Strains

**DOI:** 10.3390/ijms25137152

**Published:** 2024-06-28

**Authors:** Paweł Chlipała, Tomasz Janeczko, Marcelina Mazur

**Affiliations:** Department of Food Chemistry and Biocatalysis, Wrocław University of Environmental and Life Sciences, Norwida 25, 50-375 Wrocław, Poland; pawel.chlipala@upwr.edu.pl (P.C.); tomasz.janeczko@upwr.edu.pl (T.J.)

**Keywords:** dihydrochalcones, chalcones, biocatalysis, *Yarrowia lipolytica*

## Abstract

4′-dihydrochalcones are secondary metabolites isolated from many medicinal plants and from the resin known as ‘dragon’s blood’. Due to their biological potential, our research objective was to determine the possibilities of using biocatalysis processes carried out in deep eutectic solvents (DESs) to obtain 4′-dihydrochalcones as a model compound. The processes were carried out in a culture of the yeast *Yarrowia lipolytica* KCh 71 and also in cultures of strains of the genera *Rhodotorula* and *Debaryomyces*. Based on the experiments carried out, an optimum process temperature of 35 °C was chosen, and the most suitable DES contained glycerol as a hydrogen bond donor (HBD). For a medium with 30% water content (DES 11), the conversion observed after 24 h exceeded 70%, while increasing the amount of water to 50% resulted in a similar level of conversion after just 1 h. A fivefold increase in the amount of added substrate resulted in a reduction in conversion, which reached 30.3%. Of the other yeast strains tested, *Rhodotorula marina* KCh 77 and *Rhodotorula rubra* KCh 4 also proved to be good biocatalysts for the bioreduction process. For these strains, the conversion reached 95.4% and 95.1%, respectively. These findings highlight the potential of yeast as a biocatalyst for the selective reduction of α,β-unsaturated ketones and the possibility of using a DESs as a reaction medium in this process.

## 1. Introduction

In the context of sustainable development, biocatalysis as a technique to obtain desired compounds has a long and established history [1,2]. One of the methods that is particularly eagerly used due to its simplicity and low cost is biotransformation, which is carried out using the whole cells of microorganisms [3,4]. For various reduction reactions, it is advantageous to use yeast strains that are known for their high oxidoreductase activity [5,6]. Additionally, the use of whole-cell biocatalysts ensures that a constant supply of redox equivalents are renewed in metabolic processes [7,8]. In the literature, many examples of the selective reduction of the carbonyl groups of ketones, diketones, and oxoesters can be found [9,10,11,12].

Chalcones can undergo several types of biotransformation depending on the substrate structure and the biocatalyst used [13,14,15]. These transformations include cyclization, hydroxylation, *O*-demethylation, and glycosidation [16,17,18,19]. The reduction of the carbonyl group present in dihydrochalcones, leading to the formation of the corresponding alcohol, has also been documented [16,20,21]. One of the possible biotransformation pathways for chalcones is the reduction of the α,β-unsaturated carbonyl system to form the corresponding dihydrochalcones. This regioselective reduction is observed with whole-cell biocatalysts such as cyanobacteria, fungi, and nonconventional yeasts acting as ene-reductases [16,20,22]. Yeasts are highly selective catalysts for this hydrogenation reaction, typically yielding dihydrochalcones as the sole product without any side products [23,24]. In addition to high catalytic activity, safety is another important aspect in the selection of a biocatalyst. For this reason, yeast strains that are used in food production are particularly popular. Therefore, we started our search for an effective biocatalyst with *Yarrowia lipolytica*, which has documented activity in reducing the C=C bond [22] and is also classified as a novel food [25,26,27].

Selective bioreduction processes of various chalcone compounds are described in the literature [22,24,28,29]. In the present work, we report on the process carried out for 4′-hydroxychalcone Chalcone and dihydrochalcone compounds with the characteristic 4′-hydroxy substitution are present in, among others, a dark-red resin called ‘dragon’s blood’ (DB). DB was widely used in traditional Chinese medicine and also by the ancient Greeks and Romans as a means of promoting wound healing, treating diarrhea, reducing fever, and as an antiviral and soothing agent for skin diseases such as eczema. Currently, numerous biological properties of DB have been confirmed, such as the ability to support wound healing and ulcers and antidiarrheal, anticancer, anti-inflammatory, and antirheumatic properties [30,31]. Many dihydrochalcones derived from DB resin have free phenolic hydroxyl groups, but for this group of compounds, the presence of a carbonyl group has a positive effect on the free radical scavenging capacity and photoprotective properties [32].

In addition, an aspect of the novelty of the research we present is the use of deep eutectic solvents as a reaction medium. DESs are considered ‘green solvents’, which are easily biodegradable, nontoxic, inexpensive, and easy to prepare, and over the past few years, they have been increasingly used in extraction processes, chemical synthesis, and biotransformation [33,34,35]. DESs are specific mixtures of two or more substances that in a certain molar ratio show a significant lowering of the melting point compared to pure starting compounds [29,36,37]. DESs usually consist of quaternary ammonium salts (hydrogen bond acceptors), such as choline chloride, and hydrogen bond donors, such as sugars, polyols, or urea. When the components of DESs are primary metabolites, such as sugars, amino acids, organic acids, or choline derivatives, these DESs are the so-called natural deep eutectic solvents (NADESs) [37]. Despite the constantly growing interest in DESs, there is very little information in the literature on their use as a medium for microbial C=C double-bond reduction. Most reports concern the reduction of carbonyl groups leading to alcohols [9,10]. Therefore, we are interested in the process carried out using these solvents. In addition, the use of DESs can contribute to process efficiency due to the increased bioavailability of the substrate [38,39].

## 2. Results and Discussion

### 2.1. Biocatalysts and the Determination of the Optimal Temperature of the Biotransformation Process

Chalcones are natural compounds that commonly occur as secondary plant metabolites, and their numerous biological activities are thoroughly documented. However, much less information is available regarding 4′-hydroxychalcones. Studies most often refer to the isolation of these compounds from plants used in traditional folk medicine or the determination of their biological activities. However, the topic of 4′-hydroxychalcone biotransformation is barely explored [24,40]. Therefore, we became interested in this group of compounds and chose a 4′-hydroxychalcone as a substrate for biotransformation processes.

*trans*-4′-hydroxychalcone (*trans*-**1**) was obtained by the Claisen–Schmidt condensation of 4-hydroxyacetophenone and benzaldehyde. The structure of *trans*-**1** was confirmed based on NMR analysis and is consistent with the literature data (Figures S1–S5) [41,42].

Specific biotransformation products depend on the structure of the chalcone substrate and the biocatalyst employed, whether whole cells or isolated enzymes are used [13,14,15]. Hydroxylation and *O*-demethylation reactions are commonly observed in filamentous fungi cultures [14,43]. Entomopathogenic filamentous fungi from the genera *Isaria* and *Beauveria* can perform *O*-methylglycosylation and hydroxylation followed by the 4-*O*-methylglycosylation of selected chalcones [15,18]. Cyclization to form flavanones is catalyzed by enzymes such as chalcone isomerase [44,45]. However, the common pathway for most microbial biocatalysts is the chemoselective reduction of the α,β-unsaturated ketone moiety to dihydrochalcones. This reaction is facilitated by whole-cell biocatalysts such as bacteria, fungi, cyanobacteria, and, predominantly, yeast, which act as ene-reductases. Considering the information mentioned above and our previous experience in *trans*-4′-hydroxychalcone biotransformation processes, the *Yarrowia lipolytica* KCh 71 strain was used as the biocatalyst of choice [23,28]. An additional advantage of using *Yarrowia lipolytica* is that in 2019, the biomass of this yeast was approved as a novel food (Commission Implementing Regulation (EU) 2019/760 of 13 May 2019). Therefore, the use of this biocatalyst is safe even in the case of biomass consumption [26,27].

**Figure 1:**
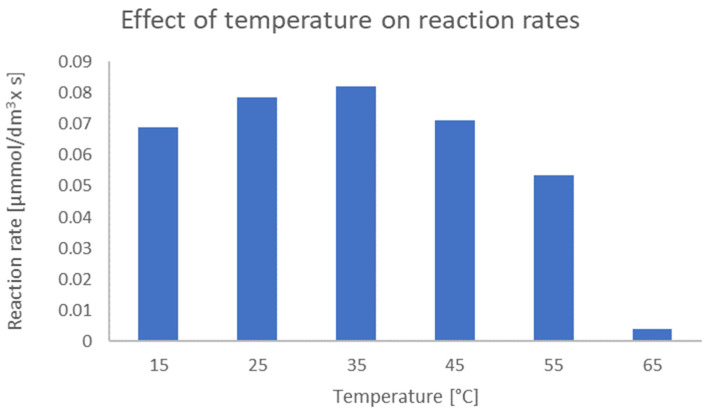
Effect of temperature on reaction rates [µmol/dm^3^ × s] after 3 h of processing in *Yarrowia lipolytica* KCh 71 culture.

The optimal temperature for the biotransformation process was selected on the basis of data obtained in a series of experiments involving the biotransformation of chalcone *trans*-**1** in a standard culture medium. The tested temperature ranged between 15 and 65 °C in increments of 10 degrees. The results of the experiments are shown in Figure 1 as reaction rates [µmol/dm^3^ × s]. The rate of a reaction was calculated as the change in the concentration of the product divided by the time during which this change occurred. The reaction was most effective at temperatures between 25 and 45 °C. A temperature of 35 °C was chosen as the optimal temperature, which is close to the optimal temperatures for the growth of *Yarrowia* strains (28 °C and 30 °C) [46,47].

**Figure 2:**
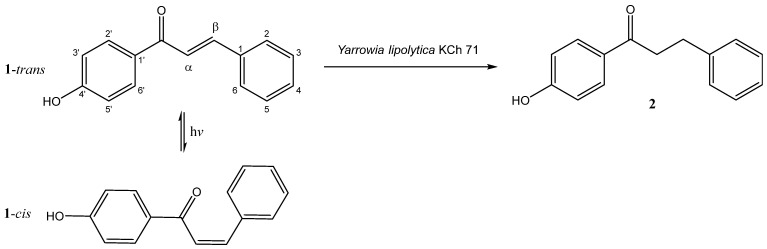
Bioreduction of chalcones in *Yarrowia lipolytica* KCh 71 culture.

As a result of the bioreduction process, dihydrochalcone **2** was obtained (Figure 2). The structure of the product was confirmed by NMR analysis. The main difference seen in the ^1^H NMR spectrum is related to the position of the signals from the H-α and H-β olefins protons, which are visible in the substrate spectrum at characteristic shifts of 7.92 and 7.68 ppm as doublets with a high coupling constant of 15.6 Hz. In the dihydrochalcone (**2**) spectrum, there is a clear shift in these signals toward the higher field, where both multiplets, with double integration, are seen as triplets (J = 7.5 Hz) at 3.23 and 2.90 ppm. A similar correlation can be observed on the ^13^C NMR spectrum (Figures S11–S15). Signals from the C-α and C-β carbon atoms in the chalcone spectrum are at a significantly lower field than in the dihydrochalcone spectrum (Table 1).

### 2.2. Use of DESs as a Medium for Selective Bioreduction Reactions

The use of deep eutectic solvents as a medium in bioprocessing is currently receiving significant attention because of the numerous benefits that their use can bring. However, since there are few reports in the literature on the bioreduction of 4′-hydroxychalcones [24], the aspect of biocatalysis performed in these types of solvent is quite niche. For this reason, once the optimum process temperature had been selected, the next aspect investigated was the effect of deep eutectic solvents on the rate of substrate conversion (with reference to GC analysis). Sixteen different NADESs and NADESs in water solutions based on choline chloride and sugars, polyols, and urea were tested. Additionally, the efficiency of the process was also tested at different water contents in deep eutectic mixtures (Table 2 and Table 3).

It should be noted that at higher water contents, mixtures are closer to an aqueous solution of individually solvated DES components. According to the literature, choline chloride–urea, commonly called reline, loses the nanostructure characteristic of a deep eutectic solvent when the addition of water reaches or exceeds 50 wt% [48]. Similarly, when DESs consisting of choline chloride and glycols were evaluated, dilution with water caused the weakening of hydrogen bonds between DES components. Gabriele et al. noticed that dilution with water caused the interactions to gradually weaken until the water added reached 50% (*w*/*w*), and at around 75% (*w*/*w*), they completely disappeared [49]. Being aware of the above phenomenon, we conducted experiments with high water addition due to the tendency for rapid substrate-to-product conversion in these mixtures. The basic correlation that can be seen for all solvents tested is the higher conversion for DES mixtures with a water content of 70%. For most of the solvents tested, with the highest addition of water, more than 80% conversions were achieved after just 1 h of the process. As the percentage of water was reduced, a decrease in the conversion rate could be observed in all media. This may be due to the inverse correlation between water content and density, as well as the viscosity of the mixtures tested [50,51]. Some authors highlight the potential that the ‘water-in-DES’ system offers to improve performance and reduce costs [52].

Analyzing the impact of the tested HBDs on substrate conversion, it can be seen that the presence of urea in the tested mixtures resulted in a significant reduction in the reaction rate compared to solvents with a similar water content. Among sugar-based DESs, the fructose-containing mixtures (DESs 4–6) turned out to be better than those containing glucose (DESs 1–3). Of the polyol-based DESs (Table 3), the reaction was faster when glycerol (DESs 10–13) was used as a component of the eutectic mixture, but when using a DES with a high water content (DES 13 and DES 16), these differences were not significant. In summary, among the DESs tested, a glycerol-based DES was chosen as the best medium. Taking into account that a higher addition of water has an inert effect on the DES nanostructure, we chose the one with 30% water (DES 11) because the process efficiency was relatively high, and after 24 h of the process, a conversion of more than 70% was obtained. For that reason, all subsequent experiments were conducted with this medium.

In addition, we also performed an experiment in which we increased the amount of substrate added. A fivefold increase in concentration (up to 1 mg/mL) resulted in a significant decrease in the rate of bioreduction, which reached only 30% after three days of the process.

During experiments with 4′-hydroxychalcone, the *trans*-chalcone isomerization to its *cis*-analogue was observed (Figure 2). The *cis*-1 chalcone was separated from the reaction mixture so that its structure was confirmed by NMR and 2D NMR analysis (Figures S6–S10). The main differences in the chemical shifts of *cis* and *trans* chalcones are apparent for the olefinic protons or in the alkyl moiety and similarly for the corresponding carbon atoms (Table 1).

We did not observe the isomerization product during the bioreduction of chalcones with a hydroxyl group at carbon C-2′, which we described in our previous publications [22,23,53]. These findings are consistent with previous observations on the photoisomerization of the thermodynamically more stable *trans*-4-hydroxychalcone carried out in different solvents by daylight irradiation. Isomerization occurred in classic organic solvents [54,55,56] (acetone, isooctane, n-pentane, methanol, benzene) as well as in water–ionic liquid biphasic systems [57].

For 2′-hydroxyalkone, the formation of an intramolecular hydrogen bond was observed between carbonyl oxygen and the hydroxyl group on C-2′ carbon [58]. Quantum yield measurements and laser flash photolysis studies confirmed that 2′-hydroxychalcone isomerizes only from the *cis* to the *trans* isomer upon photoexcitation and not in the reverse direction [59]. Therefore, under biotransformation conditions with light exposure, *trans–cis* isomerization products were not observed. Matsushima et al. reported that *trans*-2′-hydroxychalcone photoirradiation gave flavanone [60]. For 2-hydroxyhalcones, a specific equilibrium was observed between the *trans*- and *cis*-isomers and the flavylium cation, influenced by light excitation and pH [61,62,63]. In contrast, the hydroxyl group present at the 2′- or 4-position in the molecule prevented *trans–cis* photoisomerization [55]. Sidhart et al. proposed the mechanism of this process for the 4,4′-dichloro-chalcone (2*E*)-1,3-bis(4-chlorophenyl)prop-2-en-1-one based on the stable molecular arrangement of the (*Z*)-chalcone network [64]. Since the biotransformations presented in this work were carried out in a bright room with access to sunlight, we also observed the photoisomerization of the substrate, which occurs from the first hour of the biotransformation. We observed a higher percentage of isomerization product in DESs with lowest water content, which may be related to slower substrate transformation by the *Y. lipolytica* strain. As a result of the rapid microbial conversion of chalcone to dihydrochalcone, the substrate was reduced faster than it had time to isomerize. This process may have influenced the rate of biotransformation, but further analyses are required to confirm these assumptions.

The next step of the research was to compare the efficiency of the bioreduction process when other yeast strains were used. The microorganisms tested belong to yeasts of the genera *Rhodotorula* and *Debaryomyces* (Table 4). To compare the bioreduction efficiency, all reactions involving other biocatalysts were carried out using DES 11 at 35 °C. Bioreduction occurred most effectively in *R. rubra* KCh 4 and *R. rubra* KCh 82, where the conversion reached, respectively, 84 and 72% after 24 h of the process.

## 3. Materials and Methods

### 3.1. Analysis

Gas chromatography (GC, Agilent Technologies 6890N instrument, Santa Clara, CA, USA) was used to monitor the progress of the reactions. GC analysis was performed using an Agilent DB-5MS capillary column (nonpolar phenyl arylene polymer 30 m × 0.32 mm × 0.25 µm) and hydrogen as carrier gas. The following analysis conditions were applied: injector 250 °C, detector (FID) 250 °C, column temperature 80–200 °C (25 °C × min^−1^), 200–300 °C (30 °C × min^−1^), 300 °C (3 min).

The purification of the products was carried out using the PuriFlash XS520Plus system and silica gel (column 30 µm Interchim, Montluçon, France).

The NMR spectra analyses were performed on a JEOL 400 MHz Year Hold Magnet spectrometer and on a Brüker Avance II 600 MHz spectrometer (Brüker, Rheinstetten, Germany) in DMSO-*d*_6_ solution. The signals of residual solvent (δ_H_ = 2.50, δ_C_ = 39.52) were used as references.

### 3.2. Chemicals

Choline chloride (≥99%) came from Acros organics (Geel, Belgium). Fructose (≥99%) came from Alfa Aesar (Haverhill, NH, USA). D-sorbitol (≥98%), glycerol (≥99.5%), benzaldehyde (≥99%), and 4-hydroxyacetophenone (99%) came from Sigma-Aldrich (St. Louis, MO, USA). Analytical grade chemicals: glucose, urea, anhydrous magnesium sulfate, and organic solvents were purchased from P.P.H. Stanlab (Lublin, Poland), Chempur (Piekary Śląskie, Poland), and POCH (Gliwice, Poland).

### 3.3. Chemical Synthesis

4′-hydroxychalcone ((*E)*-1-(4-hydroxyphenyl)-3-phenylprop-2-en-1-one) was obtained by the Claisen–Schmidt condensation of an appropriate 4-hydroxyacetophenone and benzaldehyde dissolved in methanol with a catalytic amount of water in an alkaline condition and at elevated temperature. The product was purified by repeated recrystallization, first from ethanol and then from hexane according to the procedure described previously [22,28]. The structures of the compounds obtained by chemical synthesis and biotransformation are shown in Figure 2. The spectral data for these compounds are consistent with the literature [41,42]:

*trans*-4′-hydroxychalcone (*trans*-**1**):

^1^H NMR (600 MHz; DMSO-d_6_) δ (ppm): 10.44 (s, 1H, C-4′-OH), 8.05–8.10 (m, 2H, H-2′, and H-6′), 7.92 (d, 1H, J = 15.6 Hz, H-α), 7.85–7.89 (m, 2H, H-2, and H-6), 7.68 (d, 1H, J = 15.6 Hz, H-β), 7.42–7.47 (m, 3H, H-3, H-4, and H-5), 6.88–6.92 (m, 2H, H-3′, and H-5′). ^13^C NMR (151 MHz, DMSO-d_6_) δ: 187.14 (C=O), 162.24 (C-4′), 142.76 (C-β), 134.91 (C-1), 131.24 (C-2′ and C-6′), 130.35 (C-4), 129.12 (C-1′), 128.92 (C-2 and C-6), 128.74 (C-3 and C-5), 122.11 (C-α), 115.41 (C-3′ and C-5′).

*cis*-4′-hydroxychalcone (*cis*-**1**):

^1^H NMR (600 MHz; DMSO-d_6_) δ (ppm): 10.48 (s, 1H, C-4′-OH), 7.81–7.83 (m, 2H, H-2′, and H-6′), 7.36–7.32 (m, 2H, H-2 and H-6), 7.23–7.28 (m, 3H, H-3, H-4, and H-5), 6.93 (d, 1H, J = 13.0 Hz, H-β), 6.80–6.84 (m, 2H, H-3′, and H-5′), 6.71 (d, 1H, J = 13.0 Hz, H-α). ^13^C NMR (151 MHz, DMSO-d_6_) δ: 192.98 (C=O), 162.55 (C-4′), 136.65 (C-β), 135.44 (C-1), 131.43 (C-2′ and C-6′), 129.05 (C-2 and C-6), 128.57 (C-4), 128.36 (C-3 and C-5), 128.28 (C-1′), 128.11 (C-α), 115.56 (C-3′ and C-5′).

4′-hydroxydihydrochalcone (**2**):

^1^H NMR (400 MHz; DMSO-d_6_) δ (ppm): 10.37 (s, 1H, C-4′-OH), 7.84–7.87 (m, 2H, H-2′, and H-6′), 7.24–7.28 (m, 4H, H-2, H-3, H-5, and H-6), 7.14–7.19 (m, 1H, H-4), 6.81–6.86 (m, 2H, H-3′, and H-5′), 3.23 (t, 2H, J = 7.5 Hz, CH2-α), 2.90 (t, 2H, J = 7.5 Hz, CH2-β). ^13^C NMR (100 MHz, DMSO-d_6_) δ: 197.41 (C=O), 162.07 (C-4′), 141.53 (C-1), 130.59 (C-2′ and C-6′), 128.48 and 128.36 (C-1′, C-2, C-3, C-5, and C-6), 125.92 (C-4), 115.31 (C-3′ and C-5′), 39.04 (C-α), 29.48 (C-β).

### 3.4. Microorganisms and Determination of the Optimal Temperature of the Biotransformation Process

*Rhodotorula mucilaginosa* IHEM18459, *Rhodotorula rubra* KCh 4, *Rhodotorula rubra* KCh 82, *Rhodotorula marina* KCh 77, *Rhodotorula glutinis* KCh 242, *Yarrowia lipolytica* KCh 71, and *Debaryomyces hansenii* MI1a were used. The microorganisms were derived from the collection of the Department of Food Chemistry and Biocatalysis (KCh), Wrocław University of Environmental and Life Sciences. The culture of the strains was carried out at 25 °C in 300 mL Erlenmeyer flasks containing 50 mL of medium (3% glucose, 0.5% peptone K, and 0.5% aminobac in distilled water).

To determine the optimal biotransformation temperature, after the biomass (*Y. lipolytica* KCh 71) had grown (48 h), the culture (1 mL) was transferred to 2 mL Eppendorf tubes, and the substrate dissolved in DMSO was added to obtain the concentration 200 µg/mL. The tube was incubated on a ThermoMixer shaker at 700 rpm. After 1, 3, 6, 24, 96 h, the products were extracted three times with ethyl acetate. The combined extracts were analyzed by gas chromatography. The results were determined from three independent experiments and presented as reaction rates. The rate of a reaction was calculated as the change in the concentration of the product divided by the time during which this change occurred and were expressed as [µmol/dm^3^ × s].

### 3.5. Preparation of DESs

The components of DES mixtures were choline chloride (ChCl) as a hydrogen bond acceptor (HBA), glucose (Glu), fructose (Fru), urea (U), glycerol (Gly), and sorbitol (S) as a hydrogen bond donor (HBD). The DES components (hydrogen bond donor, hydrogen bond acceptor, and water) were placed in a 250 mL flask and stirred at 60 °C until a homogeneous and transparent liquid was formed (Table 5).

### 3.6. Biotransformation in DESs

The propagation of the microorganisms was carried out at 25 °C in 300 mL Erlenmeyer flasks containing 50 mL of medium (3% glucose, 0.5% peptone K, and 0.5% aminobac in distilled water). The biomass (1 mL) was transferred to 2 mL Eppendorf tubes and centrifuged. The biomass was washed with distilled water and centrifuged again, and then 1 mL of the appropriate DES and substrate dissolved in DMSO (final concentration in the Eppendorf tube: 200 µg/mL) was added. The tube was incubated on a ThermoMixer shaker at 800 rpm. After 1, 3, 6, 24, 96 h, the products were extracted three times with ethyl acetate. The combined extracts were dried with anhydrous magnesium sulfate and analyzed by gas chromatography.

## 4. Conclusions

In this study, we evaluated the possibility of the application of various DESs and DES-in-water solutions as a medium for the bioreduction of 4′-hydroxychalcone (*trans*-**1**). Experiments were carried out using the Yarrowia lipolytica KCh 71 strain as a biocatalyst. The first step was to determine the optimal temperature for conducting the experiments, which was checked in the range of 15–65 °C. The highest conversion was observed at 35 °C, which was therefore chosen for the following experiments. Among the variants of reactions tested, the most advantageous was the use of ChCl:Gly mixtures. For a medium containing 30% water (DES 11), up to 70% conversion was observed after 24 h. However, a pronounced tendency for conversion to increase with increasing amounts of water in the reaction medium could be observed. For variants with 70% water, conversion exceeded 90% after just 24 h of the process. Of the DESs tested, the lowest conversion was observed when urea was used as the HBD. We also noticed that increasing the amount of added substrate caused a significant decrease in conversion. In all variants of the experiments, the *trans*-**1** chalcone photoisomerization process was observed simultaneously with the hydrogenation process. Among the other yeast strains tested, R. marina KCh 77 and R. rubra KCh 4 were effective biocatalysts for the process.

## Figures and Tables

**Table 1:** The ^1^H-NMR chemical shifts (ppm) of *trans* and *cis* chalcones and dihydrochalcone.

	4′-Hydroxychalcone		4′-Hydroxydihydrochalcone	
	*trans*-1	*cis*-1		2
H-α	7.92 d, *J* = 15.6 Hz	6.71 d, *J* = 13.0 Hz	CH_2_-α	3.23 t, *J* = 7.5 Hz
H-β	7.68 d, *J* = 15.6 Hz	6.93 d, *J* = 13.0 Hz	CH_2_-β	2.90 t, *J* = 7.5 Hz
C-α	122.11	128.11		39.04
C-β	142.76	136.65		29.48

**Table 2:** Composition (in % according to GC) of the product mixtures in the biotransformations of chalcone **1** in *Yarrowia lipolytica* KCh 71 culture with the use of DESs 1–9 as a medium.

	DES 1	DES 2	DES 3	DES 4	DES 5	DES 6	DES 7	DES 8	DES 9
1 h									
*cis*-**1**	20.0 ± 0.2	18.8 ± 1.5	12.7 ± 0.9	13.7 ± 2.8	7.8 ± 0.8	0.5 ± 0.2	5.4 ± 0.4	3.9 ± 0.4	6.2 ± 1.1
*trans*-**1**	70.8 ± 4.1	60.7 ± 5.3	3.2 ± 0.7	78.2 ± 2.0	40.2 ± 3.9	0.2 ± 0.2	79.8 ± 0.2	85.4 ± 0.7	79.0 ± 1.6
**2**	9.2 ± 4.0	20.5 ± 6.7	84.1 ± 0.9	8.1 ± 4.8	51.9 ± 4.6	99.3 ± 0.3	14.8 ± 0.5	10.7 ± 0.3	14.7 ± 0.6
3 h									
*cis* **-1**	22.5 ± 2.5	18.1 ± 1.7	9.5 ± 1.1	18.8 ± 1.5	1.1 ± 0.4	0.0	4.8 ± 0.3	3.7 ± 0.1	5.0 ± 0.5
*trans*-**1**	61.2 ± 5.6	37.4 ± 8.3	3.9 ± 0.2	65.5 ± 5.4	1.2 ± 0.4	0.0	84.7 ± 1.3	84.1 ± 1.7	75.4 ± 1.3
**2**	16.3 ± 6.6	44.4 ± 9.0	86.5 ± 0.9	15.7 ± 5.0	97.8 ± 0.8	100.0	10.5 ± 1.4	12.3 ± 1.7	19.6 ± 0.9
6 h									
*cis*-**1**	62.5 ± 3.5	41.7 ± 4.3	7.7 ± 0.4	22.7 ± 1.4	0.0	0.0	20.7 ± 1.7	18.4 ± 1.1	17.3 ± 4.6
*trans*-**1**	26.9 ± 2.2	15.0 ± 5.3	3.6 ± 0.4	64.4 ± 0.8	0.0	0.0	67.5 ± 2.7	69.9 ± 0.6	63.5 ± 0.7
**2**	10.5 ± 4.6	43.2 ± 9.2	88.7 ± 0.6	12.8 ± 12.8	100.0	100.0	11.8 ± 1.1	11.7 ± 1.1	19.2 ± 4.2
24 h									
*cis*-**1**	29.2 ± 1.8	22.6 ± 1.5	3.7 ± 0.6	37.5 ± 2.3	0.3 ± 0.1	0.1 ± 0.1	31.4 ± 5.4	25.8 ± 4.3	23.0 ± 3.6
*trans*-**1**	54.6 ± 2.7	25.2 ± 0.6	3.2 ± 0.8	34.7 ± 2.1	0.7 ± 0.4	0.2 ± 0.0	55.6 ± 3.6	61.6 ± 5.2	61.0 ± 4.9
**2**	16.2 ± 1.2	52.4 ± 2.2	93.1 ± 1.4	27.8 ± 2.7	99.0 ± 0.4	99.7 ± 0.1	13.1 ± 2.1	12.6 ± 1.0	16.0 ± 3.0
96 h									
*cis*-**1**	32.5 ± 5.5	15.4 ± 0.2	1.7 ± 0.3	22.3 ± 0.9	0.0	0.0	36.1 ± 2.4	32.8 ± 4.4	20.0 ± 3.3
*trans*-**1**	38.7 ± 2.3	25.6 ± 2.6	2.7 ± 0.9	43.2 ± 3.4	2.1 ± 1.3	0.6 ± 0.1	49.9 ± 2.7	53.8 ± 4.6	42.2 ± 0.7
**2**	28.9 ± 7.6	59.0 ± 2.7	95.6 ± 0.7	34.5 ± 2.5	97.9 ± 1.3	99.40.1	14.0 ± 2.7	13.4 ± 0.2	37.8 ± 4.0

**Table 3:** Composition (in % according to GC) of the product mixtures in the biotransformations of chalcone **1** in *Yarrowia lipolytica* KCh 71 culture with the use of DESs 10–16 as a medium.

	DES 10	DES 11	* DES 11 100 mg	DES 12	DES 13	DES 14	DES 15	DES 16
1 h								
*cis*-**1**	9.1 ± 3.6	7.7 ± 0.1	9.9 ± 0.7	4.8 ± 2.7	0.0	9.1 ± 1.4	5.5 ± 0.8	0.3 ± 0.1
*trans*-**1**	70.4 ± 12.0	77.0 ± 2.0	86.1 ± 1.7	25.9 ± 3.1	2.7 ± 2.3	78.1 ± 1.3	66.8 ± 1.4	2.3 ± 1.6
**2**	20.5 ± 8.4	15.3 ± 2.1	4.0 ± 1.0	71.0 ± 3.5	97.3 ± 2.3	12.8 ± 2.7	27.7 ± 1.0	97.4 ± 1.6
3 h								
*cis*-**1**	14.3 ± 3.6	7.6 ± 0.5	11.0 ± 0.9	0.7 ± 0.1	0.1 ± 0.1	18.2 ± 3.7	4.8 ± 1.7	0.0
*trans*-**1**	71.3 ± 11.3	56.9 ± 4.0	80.4 ± 3.3	2.8 ± 1.4	2.1 ± 1.8	62.2 ± 8.6	22.5 ± 6.3	1.2 ± 0.8
**2**	12.0 ± 4.1	35.5 ± 3.6	8.6 ± 2.4	96.5 ± 1.4	97.8 ± 1.9	19.6 ± 4.9	72.7 ± 7.9	98.8 ± 0.8
6 h								
*cis*-**1**	19.4 ± 6.0	5.4 ± 1.0	3.2 ± 0.3	0.5 ± 0.1	0.0	10.7 ± 0.8	2.8 ± 0.5	0.0
*trans*-**1**	68.1 ± 9.5	48.0 ± 5.6	83.8 ± 2.2	1.6 ± 1.1	2.0 ± 1.4	61.6 ± 7.2	4.3 ± 0.6	0.6 ± 0.2
**2**	12.5 ± 4.2	46.6 ± 5.5	13.0 ± 2.4	97.8 ± 1.2	98.0 ± 1.4	27.7 ± 6.4	92.9 ± 0.2	99.4 ± 0.2
24 h								
*cis*-**1**	16.1 ± 4.1	3.8 ± 1.2	2.9 ± 0.3	0.2 ± 0.1	0.0	22.6 ± 2.5	2.5 ± 0.4	0.0
*trans*-**1**	68.3 ± 7.8	23.2 ± 5.8	75.1 ± 6.7	1.6 ± 1.2	0.1 ± 0.1	41.4 ± 7.6	3.7 ± 1.1	0.6 ± 0.2
**2**	15.6 ± 3.2	73.0 ± 7.3	22.0 ± 7.0	98.3 ± 1.2	99.9 ± 0.1	36 ± 5.1	93.8 ± 0.9	99.4 ± 0.2
96 h								
*cis*-**1**	40.2 ± 3.9	3.6 ± 0.6	4.0 ± 0.2	0.0	0.0	29.4 ± 0.3	1.2 ± 0.2	0.3 ± 0.1
*trans*-**1**	44.0 ± 1.0	8.6 ± 4.4	65.8 ± 2.1	1.3 ± 0.4	2.7 ± 1.8	28.5 ± 3.1	1.0 ± 0.7	0.3 ± 0.1
**2**	15.9 ± 3.0	87.6 ± 3.8	30.3 ± 1.9	98.7 ± 0.4	97.3 ± 1.8	42.1 ± 2.8	97.8 ± 0.8	99.4 ± 0.2

* Experiment with increased substrate concentration—final concentration in Eppendorf tube: 1 mg/mL.

**Table 4:** Composition (in % according to GC) of the product mixtures in the biotransformations of chalcone **1** in yeast.

	*R. rubra* KCh 4	*R. mucilaginosa* IHEM18459	*R. marina* KCh 77	*R. rubra* KCh 82	*R. glutinis* KCh 242	*D. hansenii* MI1a
1 h						
*cis* **-1**	4.6 ± 0.7	14.0 ± 0.5	10.1 ± 3.0	5.8 ± 0.4	13.0 ± 1.1	6.6 ± 0.3
*trans* **-1**	85.2 ± 1.0	77.4 ± 2.2	72.6 ± 2.7	89.9 ± 0.5	85.2 ± 1.3	91.5 ± 0.6
**2**	10.2 ± 1.5	8.5 ± 2.6	17.2 ± 2.0	4.3 ± 0.2	1.8 ± 0.2	1.9 ± 0.3
3 h						
*cis* **-1**	7.2 ± 0.3	15.6 ± 2.1	9.6 ± 3.3	12.9 ± 0.4	18.4 ± 2.4	12.3 ± 1.5
*trans* **-1**	64.1 ± 1.3	59.5 ± 2.6	50.2 ± 5.0	71.6 ± 0.7	78.7 ± 2.6	84.1 ± 1.7
**2**	28.7 ± 1.0	24.9 ± 3.9	40.1 ± 7.5	15.5 ± 1.0	2.9 ± 0.2	3.6 ± 0.2
6 h						
*cis* **-1**	13.5 ± 0.4	17.0 ± 0.7	9.6 ± 3.8	17.9 ± 2.1	22.5 ± 1.5	23.0 ± 1.3
*trans* **-1**	46.2 ± 2.1	45.0 ± 4.7	14.2 ± 3.1	57.6 ± 3.3	74.3 ± 1.0	69.6 ± 2.1
**2**	40.3 ± 1.9	37.9 ± 4.9	76.2 ± 6.9	24.5 ± 5.1	3.3 ± 0.6	7.4 ± 0.9
24 h						
*cis* **-1**	7.2 ± 1.7	10.4 ± 1.4	4.6 ± 1.6	18.2 ± 5.1	16.6 ± 1.3	35.1 ± 2.5
*trans* **-1**	8.5 ± 3.2	26.3 ± 6.5	1.1 ± 0.7	9.8 ± 3.3	80.0 ± 2.3	46.6 ± 7.8
**2**	84.3 ± 4.8	63.3 ± 7.2	94.3 ± 2.7	72.0 ± 8.4	3.4 ± 1.0	18.2 ± 5.9
96 h						
*cis* **-1**	3.0 ± 1.4	11.8 ± 4.9	1.9 ± 0.3	11.7 ± 0.7	32.4 ± 2.2	37.3 ± 1.3
*trans* **-1**	1.9 ± 1.0	12.8 ± 5.2	2.7 ± 0.3	2.0 ± 0.1	62.1 ± 3.8	38.3 ± 2.3
**2**	95.1 ± 1.7	75.5 ± 10.1	95.4 ± 0.2	86.3 ± 0.7	5.4 ± 1.9	24.3 ± 2.5

**Table 5:** List of the DESs used in this study. Hydrogen bond acceptor was choline chloride for all DES solutions. Water content (%, *w*/*w*).

DES	1	2	3		4	5	6		7	8	9		10	11	12	13		14	15	16
% H_2_O	30	50	70		30	50	70		10	30	50		10	30	50	70		30	50	70
HBD	glucose				fructose				urea				glycerol					sorbitol		
Molar ratio HBA:HBD	2:1				1.9:1				1:2				1:2					1:1		

## Data Availability

Data is contained within the article and Supplementary Material.

## Supplementary Materials

The following supporting information can be downloaded at: https://www.mdpi.com/article/10.3390/ijms25137152/s1.

## References

[1] Bornscheuer U.T., Bucholz K. (2005). Highlights in biocatalysis—Historical landmarks and current trends. Eng. Life Sci..

[2] Pyser J.B., Chakrabarty S., Romero E.O., Narayan A.R.H. (2021). State-of-the-Art Biocatalysis. ACS Cent. Sci..

[3] de Oliveira N.S., da Silva G.P.L., Furlan O., Peña L.C., Bianchini L.F., Parahitiyawa N., Rosa E.A.R. (2023). The song remains the same. The lab bench dilemma of using shaken flasks in microbial biotransformation experiments. Biocatal. Biotransformation.

[4] Garzón-Posse F., Becerra-Figueroa L., Hernández-Arias J., Gamba-Sánchez D. (2018). Whole Cells as Biocatalysts in Organic Transformations. Molecules.

[5] Crnoglavac Popović M., Stanišić M., Prodanović R. (2024). State of the Art Technologies for High Yield Heterologous Expression and Production of Oxidoreductase Enzymes: Glucose Oxidase, Cellobiose Dehydrogenase, Horseradish Peroxidase, and Laccases in Yeasts *P. pastoris* and *S. cerevisiae*. Fermentation.

[6] Fu X., Hong K., Wang H., Zhang C., Lu W. (2022). Screening and Remodeling of Enone Oxidoreductase for High Production of 2(or 5)-Ethyl-5(or 2)-methyl-4-hydroxy-3(2H)-Furanone in Saccharomyces Cerevisiae. J. Agric. Food Chem..

[7] de Gonzalo G., Alcántara A.R. (2021). Multienzymatic Processes Involving Baeyer–Villiger Monooxygenases. Catalysts.

[8] Zappaterra F., Costa S., Summa D., Bertolasi V., Semeraro B., Pedrini P., Buzzi R., Vertuani S. (2021). Biotransformation of Cortisone with *Rhodococcus rhodnii*: Synthesis of New Steroids. Molecules.

[9] Panić M., Delač D., Roje M., Radojčić Redovniković I., Cvjetko Bubalo M. (2019). Green asymmetric reduction of acetophenone derivatives: Saccharomyces cerevisiae and aqueous natural deep eutectic solvent. Biotechnol. Lett..

[10] Cvjetko Bubalo M., Mazur M., Radošević K., Radojčić Redovniković I. (2015). Baker’s yeast-mediated asymmetric reduction of ethyl 3-oxobutanoate in deep eutectic solvents. Process. Biochem..

[11] Csuka P., Nagy-Győr L., Molnár Z., Paizs C., Bódai V., Poppe L. (2021). Characterization of Yeast Strains with Ketoreductase Activity for Bioreduction of Ketones. Period. Polytech. Chem. Eng..

[12] Monna T., Fuhshuku K.I. (2020). Biocatalytic reductive desymmetrization of prochiral 1,3-diketone and its application to microbial hormone synthesis. Mol. Catal..

[13] Stompor M., Broda D., Bajek-Bil A. (2019). Dihydrochalcones: Methods of Acquisition and Pharmacological Properties—A First Systematic Review. Molecules.

[14] Aguiar L.O., Silva E.D.O., David J.M. (2022). Biotransformation of chalcones and flavanones: An update on their bio-based derivatizations. Biocatal. Biotransformation.

[15] Krawczyk-Łebek A., Dymarska M., Janeczko T., Kostrzewa-Susłow E. (2021). New Glycosylated Dihydrochalcones Obtained by Biotransformation of 2′-Hydroxy-2-methylchalcone in Cultures of Entomopathogenic Filamentous Fungi. Int. J. Mol. Sci..

[16] Żyszka B., Anioł M., Lipok J. (2017). Highly effective, regiospecific reduction of chalcone by cyanobacteria leads to the formation of dihydrochalcone: Two steps towards natural sweetness. Microb. Cell Fact..

[17] de Matos I.L., Nitschke M., Porto A.L.M. (2023). Regioselective and chemoselective biotransformation of 2′-hydroxychalcone derivatives by marine-derived fungi. Biocatal. Biotransformation.

[18] Chlipała P., Tronina T., Dymarska M., Urbaniak M., Kozłowska E., Stępień Ł., Kostrzewa-Susłow E., Janeczko T. (2024). Multienzymatic biotransformation of flavokawain B by entomopathogenic filamentous fungi: Structural modifications and pharmacological predictions. Microb. Cell Fact..

[19] Krawczyk-Łebek A., Dymarska M., Janeczko T., Kostrzewa-susłow E. (2022). Glycosylation of Methylflavonoids in the Cultures of Entomopathogenic Filamentous Fungi as a Tool for Obtaining New Biologically Active Compounds. Int. J. Mol. Sci..

[20] Kozłowska J., Potaniec B., Anioł M. (2020). Biotransformation of Hydroxychalcones as a Method of Obtaining Novel and Unpredictable Products Using Whole Cells of Bacteria. Catalysts.

[21] Stompor M., Kałużny M., Żarowska B. (2016). Biotechnological methods for chalcone reduction using whole cells of *Lactobacillus*, *Rhodococcus* and *Rhodotorula* strains as a way to produce new derivatives. Appl. Microbiol. Biotechnol..

[22] Łużny M., Kaczanowska D., Gawdzik B., Wzorek A., Pawlak A., Obmińska-Mrukowicz B., Dymarska M., Kozłowska E., Kostrzewa-Susłow E., Janeczko T. (2022). Regiospecific Hydrogenation of Bromochalcone by Unconventional Yeast Strains. Molecules.

[23] Łużny M., Kozłowska E., Kostrzewa-Susłow E., Janeczko T. (2020). Methoxychalcone by *Yarrowia lipolytica* Enables. Catalysts.

[24] Filippucci S., Tasselli G., Kenza Labbani F.Z., Turchetti B., Rita Cramarossa M., Buzzini P., Forti L. (2020). Non-conventional yeasts as sources of ene-reductases for the bioreduction of chalcones. Fermentation.

[25] Zieniuk B., Jasińska K., Wierzchowska K., Uğur Ş., Fabiszewska A. (2024). *Yarrowia lipolytica* Yeast: A Treasure Trove of Enzymes for Biocatalytic Applications—A Review. Fermentation.

[26] Turck D., Castenmiller J., de Henauw S., Hirsch-Ernst K., Kearney J., Maciuk A., Mangelsdorf I., McArdle H.J., Naska A., Pelaez C. (2019). Safety of *Yarrowia lipolytica* yeast biomass as a novel food pursuant to Regulation (EU) 2015/2283. EFSA J..

[27] Turck D., Bohn T., Castenmiller J., De Henauw S., Hirsch-Ernst K.I., Maciuk A., Mangelsdorf I., McArdle H.J., Naska A., Pelaez C. (2022). Safety of an extension of use of *Yarrowia lipolytica* yeast biomass as a novel food pursuant to Regulation (EU) 2015/2283. EFSA J..

[28] Łuzny M., Krzywda M., Kozłowska E., Kostrzewa-Susłow E., Janeczko T. (2019). Effective Hydrogenation of 3-(2”-furyl)- And 3-(2”-thienyl)-1-(2′-hydroxyphenyl)-prop-2-en-1-one in Selected Yeast Cultures. Molecules.

[29] Perna F.M., Vitale P., Capriati V. (2020). Deep eutectic solvents and their applications as green solvents. Curr. Opin. Green Sustain. Chem..

[30] Gupta D., Gupta R.K. (2011). Bioprotective properties of Dragon’s blood resin: In vitro evaluation of antioxidant activity and antimicrobial activity. BMC Complement. Altern. Med..

[31] Al-Awthan Y.S., Bahattab O.S. (2021). Phytochemistry and Pharmacological Activities of Dracaena cinnabari Resin. Biomed. Res. Int..

[32] Wang S., Shi Y., Ma J., Ye Z., Yao M., Shang J., Liu J. (2022). Enhanced intradermal delivery of Dragon’s blood in biocompatible nanosuspensions hydrogel patch for skin photoprotective effect. J. Cosmet. Dermatol..

[33] Mazur M., Janeczko T., Gładkowski W. (2022). Lipase-mediated Baeyer-Villiger oxidation of benzylcyclopentanones in ester solvents and deep eutectic solvents. Sci. Rep..

[34] Grudniewska A., Popłoński J. (2020). Simple and green method for the extraction of xanthohumol from spent hops using deep eutectic solvents. Sep. Purif. Technol..

[35] Di Carmine G., Abbott A.P., D’Agostino C. (2021). Deep eutectic solvents: Alternative reaction media for organic oxidation reactions. React. Chem. Eng..

[36] Pätzold M., Siebenhaller S., Kara S., Liese A., Syldatk C., Holtmann D. (2019). Deep Eutectic Solvents as Efficient Solvents in Biocatalysis. Trends Biotechnol..

[37] Paiva A., Craveiro R., Aroso I., Martins M., Reis R.L., Duarte A.R.C. (2014). Natural deep eutectic solvents—Solvents for the 21st century. ACS Sustain. Chem. Eng..

[38] Panić M., Hrvat N.M., Štokić M., Radojčić Redovniković I., Kovarik Z., Radošević K. (2022). Natural deep eutectic solvents improve the solubility of acetylcholinesterase reactivator RS194B. Sustain. Chem. Pharm..

[39] Yang T.X., Zhao L.Q., Wang J., Song G.L., Liu H.M., Cheng H., Yang Z. (2017). Improving Whole-Cell Biocatalysis by Addition of Deep Eutectic Solvents and Natural Deep Eutectic Solvents. ACS Sustain. Chem. Eng..

[40] Nawade B., Yahyaa M., Davidovich-Rikanati R., Lewinsohn E., Ibdah M. (2020). Optimization of Culture Conditions for the Efficient Biosynthesis of Trilobatin from Phloretin by Engineered Escherichia coli Harboring the Apple Phloretin-4′-O-glycosyltransferase. J. Agric. Food Chem..

[41] Dimmock J.R., Murthi Kandepu N., Hetherington M., Wilson Quail J., Pugazhenthi U., Sudom A.M., Chamankhah M., Rose P., Pass E., Allen T.M. (1998). Cytotoxic activities of Mannich bases of chalcones and related compounds. J. Med. Chem..

[42] Vitali A., Giardina B., Delle Monache G., Rocca F., Silvestrini A., Tafi A., Botta B. (2004). Chalcone dimethylallyltransferase from Morus nigra cell cultures. Substrate specificity studies. FEBS Lett..

[43] Sanchez-Gonzalez M., Rosazza J.P.N. (2004). Microbial Transformations of Chalcones: Hydroxylation, O -Demethylation, and Cyclization to Flavanones. J. Nat. Prod..

[44] Lewis J.A., Jacobo E.P., Palmer N., Vermerris W., Sattler S.E., Brozik J.A., Sarath G., Kang C. (2024). Structural and Interactional Analysis of the Flavonoid Pathway Proteins: Chalcone Synthase, Chalcone Isomerase and Chalcone Isomerase-like Protein. Int. J. Mol. Sci..

[45] Furumura S., Ozaki T., Sugawara A., Morishita Y., Tsukada K., Ikuta T., Inoue A., Asai T. (2023). Identification and Functional Characterization of Fungal Chalcone Synthase and Chalcone Isomerase. J. Nat. Prod..

[46] Jach M.E., Malm A. (2022). *Yarrowia lipolytica* as an Alternative and Valuable Source of Nutritional and Bioactive Compounds for Humans. Molecules.

[47] López-Trujillo J., Mellado-Bosque M., Ascacio-Valdés J.A., Prado-Barragán L.A., Hernández-Herrera J.A., Aguilera-Carbó A.F. (2023). Temperature and pH Optimization for Protease Production Fermented by *Yarrowia lipolytica* from Agro-Industrial Waste. Fermentation.

[48] Hammond O.S., Bowron D.T., Edler K.J. (2017). The Effect of Water upon Deep Eutectic Solvent Nanostructure: An Unusual Transition from Ionic Mixture to Aqueous Solution. Angew. Chem..

[49] Gabriele F., Chiarini M., Germani R., Tiecco M., Spreti N. (2019). Effect of water addition on choline chloride/glycol deep eutectic solvents: Characterization of their structural and physicochemical properties. J. Mol. Liq..

[50] Yadav A., Pandey S. (2014). Densities and viscosities of (choline chloride + urea) deep eutectic solvent and its aqueous mixtures in the temperature range 293.15 K to 363.15 K. J. Chem. Eng. Data.

[51] Moghimi M., Roosta A. (2019). Physical properties of aqueous mixtures of (choline chloride + glucose) deep eutectic solvents. J. Chem. Thermodyn..

[52] López-Salas N., Vicent-Luna J.M., Imberti S., Posada E., Roldán M.J., Anta J.A., Balestra S.R.G., Madero Castro R.M., Calero S., Jiménez-Riobóo R.J. (2019). Looking at the “water-in-Deep-Eutectic-Solvent” System: A Dilution Range for High Performance Eutectics. ACS Sustain. Chem. Eng..

[53] Janeczko T., Gładkowski W., Kostrzewa-Susłow E. (2013). Microbial transformations of chalcones to produce food sweetener derivatives. J. Mol. Catal. B Enzym..

[54] Lutz R.T., Jordan R.H. (1950). cis-Benzalacetophenone. J. Am. Chem. Soc..

[55] Iwata S., Nishino T., Inoue H., Nagata N., Satomi Y., Nishino H., Shibata S. (1997). Antitumorigenic activity of chalcones (II). Photo-isomerization of chalcones and correlation with their biological activities. Biol. Pharm. Bull..

[56] Baas P., Cerfontain H. (1977). Conformational study on some β-phenyl-α,β-unsaturated ketones. Tetrahedron.

[57] Fernandez D., Parola A.J., Branco L.C., Afonso C.A.M., Pina F. (2004). Thermal and photochemical properties of 4′-hydroxyflavylium in water-ionic liquid biphasic systems. J. Photochem. Photobiol. A Chem..

[58] Norikane Y., Itoh H., Arai T. (2002). Photochemistry of 2′-hydroxychalcone. One-way cis-trans photoisomerization induced by adiabatic intramolecular hydrogen atom transfer. J. Phys. Chem. A.

[59] Norikane Y., Itoh H., Arai T. (2000). Control of the photoisomerization mode of carbon-carbon double bond by intramolecular hydrogen bond; one-way photoisomerization of 2′-hydroxychalcone induced by adiabatic intramolecular hydrogen atom transfer. Chem. Lett..

[60] Matsushima R., Kageyama H. (1985). Photochemical cyclization of 2′-hydroxychalcones. J. Chem. Soc. Perkin Trans..

[61] Roque A., Lima J.C., Parola A.J., Pina F. (2007). Substitution and solvent effects in the chalcones isomerization barrier of flavylium photochromic systems. Photochem. Photobiol. Sci..

[62] Kalchevski D.A., Petrov V., Tadjer A., Nenov A. (2018). Impacts of hydroxylation on the photophysics of chalcones: Insights into the relation between the chemical composition and the electronic structure. Phys. Chem. Chem. Phys..

[63] Pina F., Roque A., Melo M.J., Maestri M., Belladelli L., Balzani V. (1998). Multistate/multifunctional molecular-level systems: Light and pH switching between the various forms of a synthetic flavylium salt. Chem. A Eur. J..

[64] Sidharth S.N., Yuvaraj A.R., Hui T.J., Sarojini B.K., Mashitah M.Y., Hegde G. (2014). Light induced properties of chalcones correlated with molecular structure and photophysical properties for permanent optical storage device. Adv. Mater. Res..

