# Supplementary material for: Bioreduction of 4′-Hydroxychalcone in Deep Eutectic Solvents: Optimization and Efficacy with Various Yeast Strains
Source: Int J Mol Sci. 2024 Jun 28;25(13):7152. doi: 10.3390/ijms25137152 (PMC11241015; doi:10.3390/ijms25137152)
Supplement: Supplementary file 1 [file ijms-25-07152-s001.zip › ijms-3071137-supplementary.pdf]

International Journal of Molecular Sciences

Supplementary Materials

Bioreduction of 4'-hydroxychalcone in deep eutectic solvents:

optimization and efficacy with various yeast strains

Paweł Chlipała, Tomasz Janeczko, Marcelina Mazur*

Department of Food Chemistry and Biocatalysis, Wrocław University of Environmental and Life Sciences, Norwida 25, 50-375 Wrocław, Poland

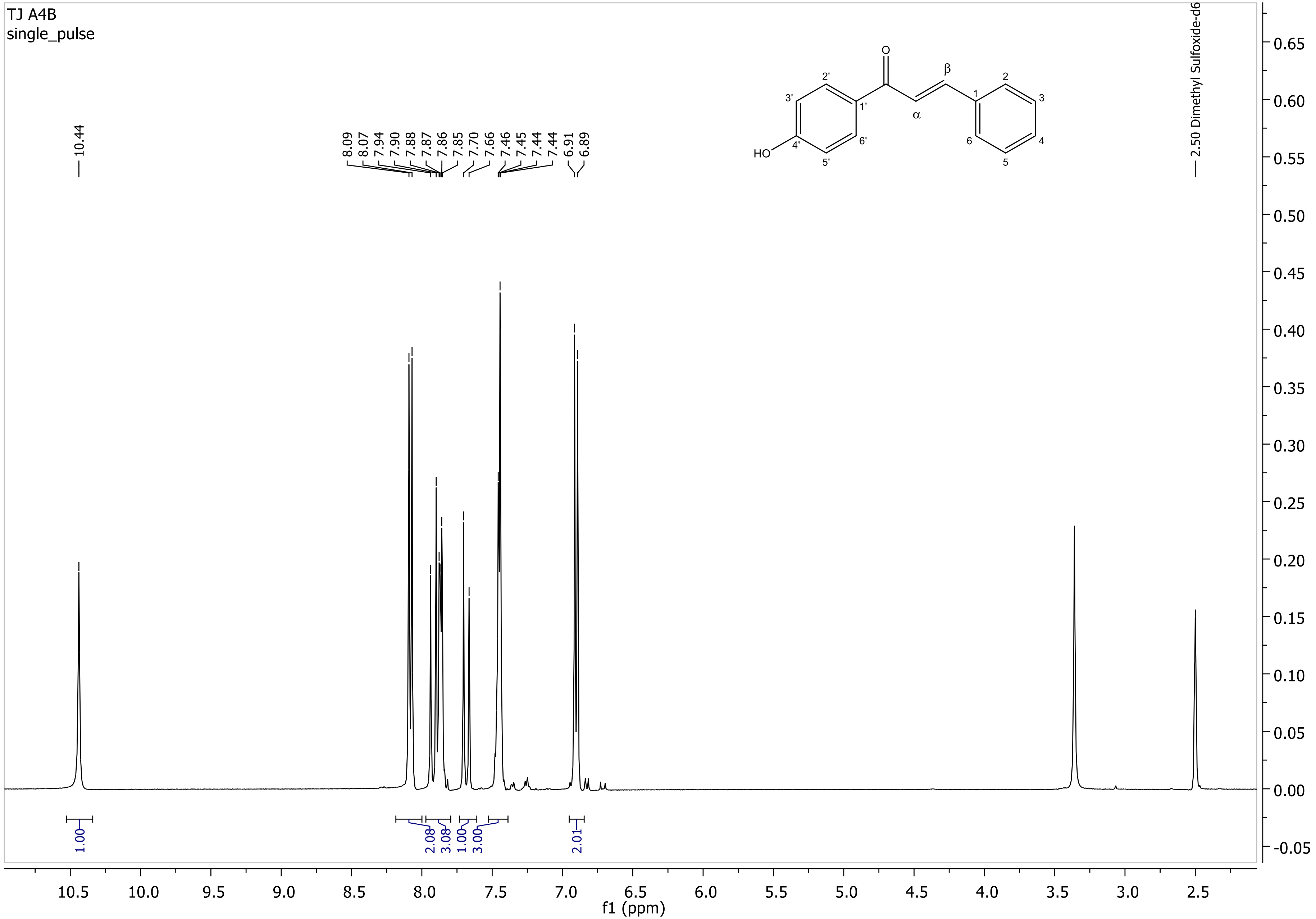

Figure S1: ^{1}H-NMR spectrum of 4'-hydroxychalcone (*trans*-**1**).

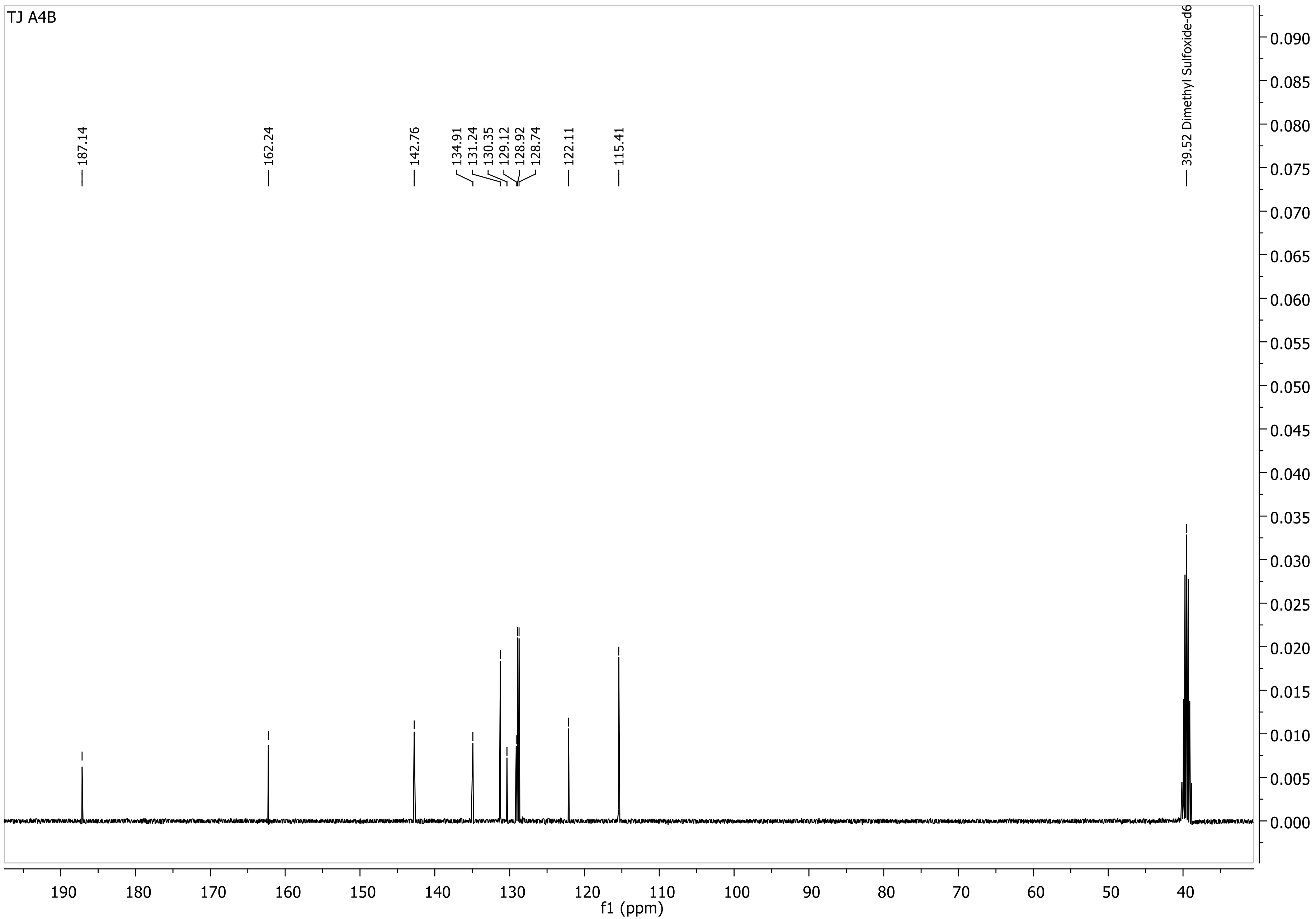

Figure S2: ^{13}C-NMR spectrum of 4'-hydroxychalcone (*trans*-**1**).

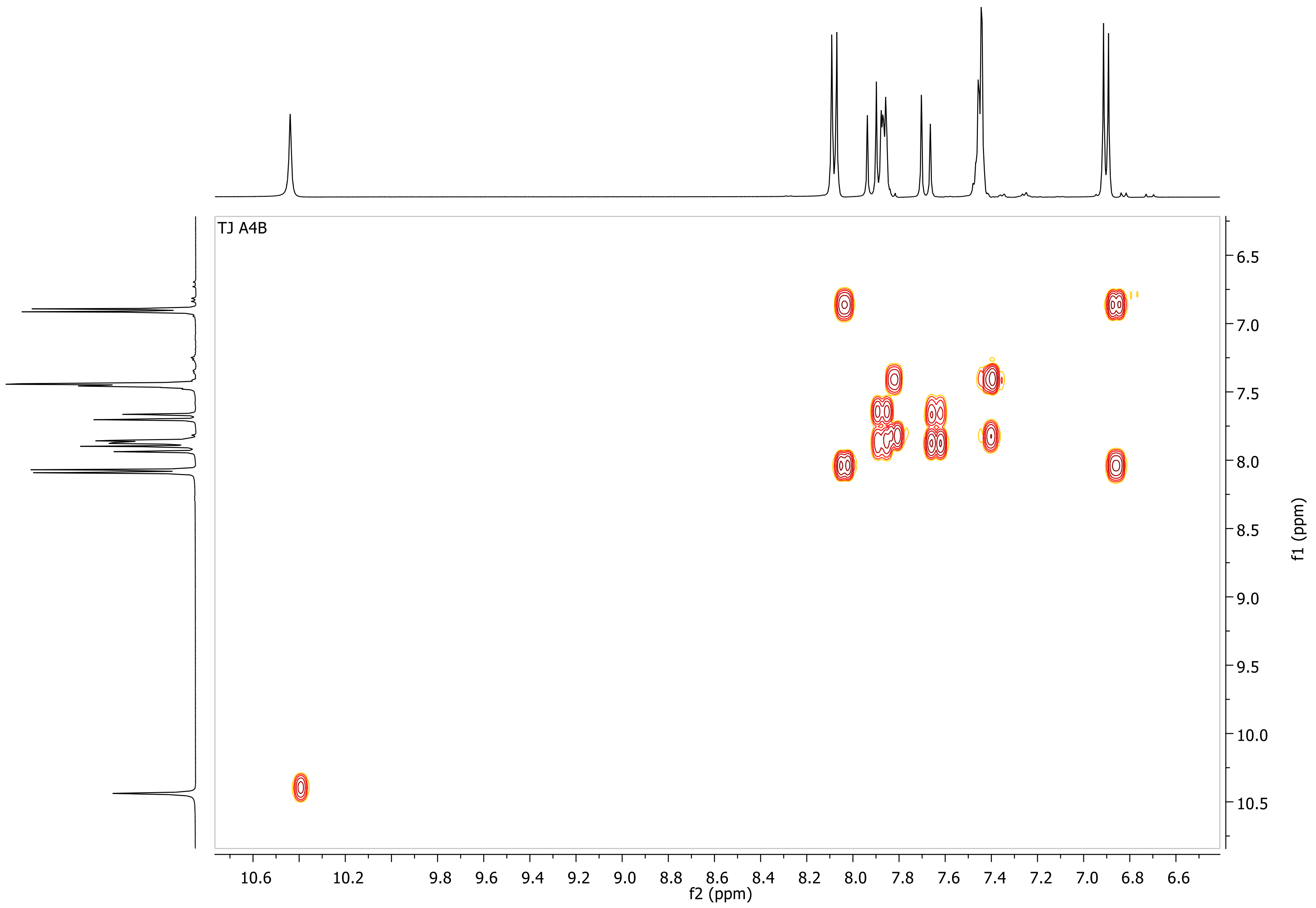

Figure S3: COSY spectrum of 4'-hydroxychalcone (*trans*-**1**).

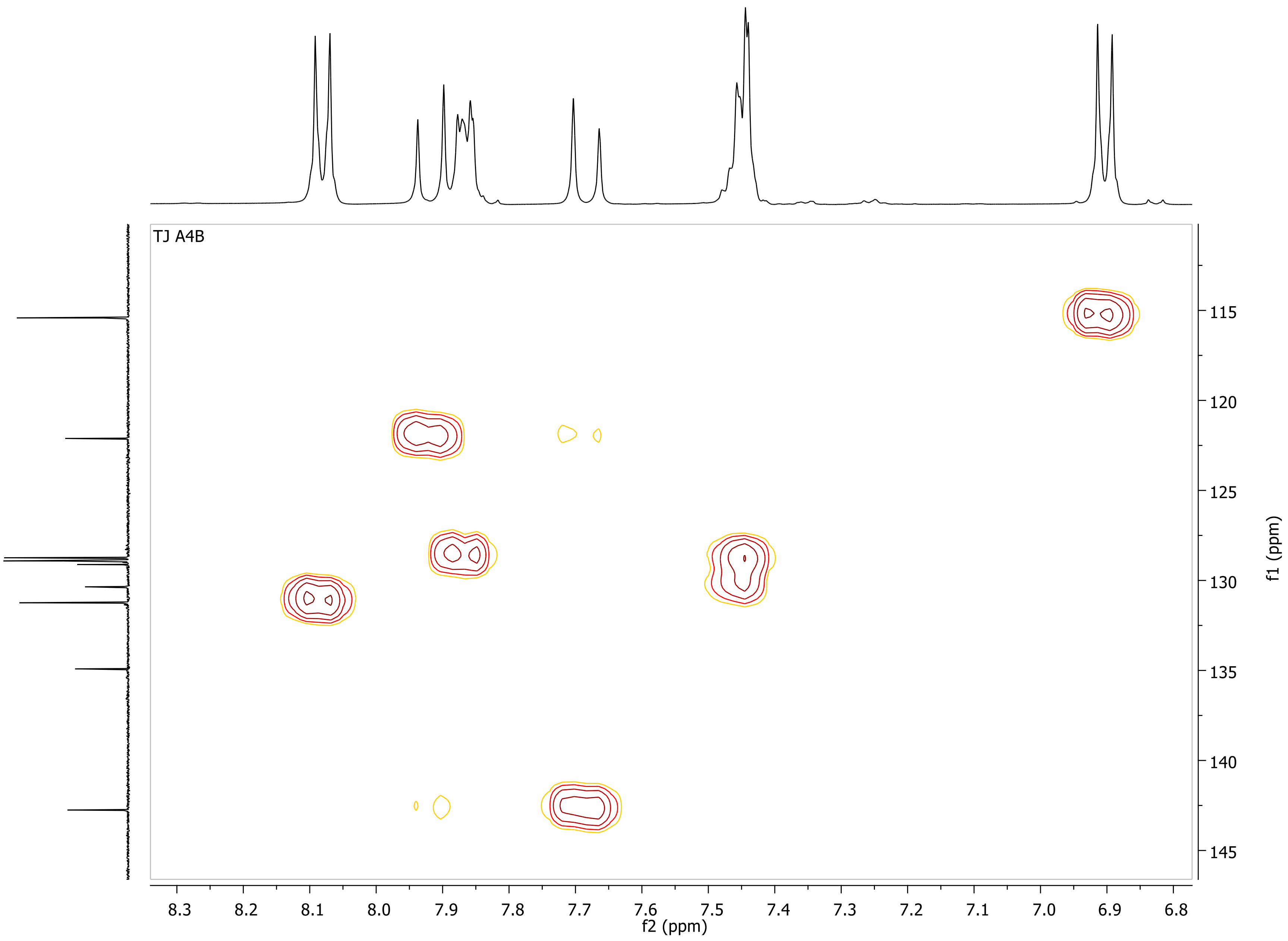

Figure S4: HSQC spectrum of 4'-hydroxychalcone (*trans*-**1**).

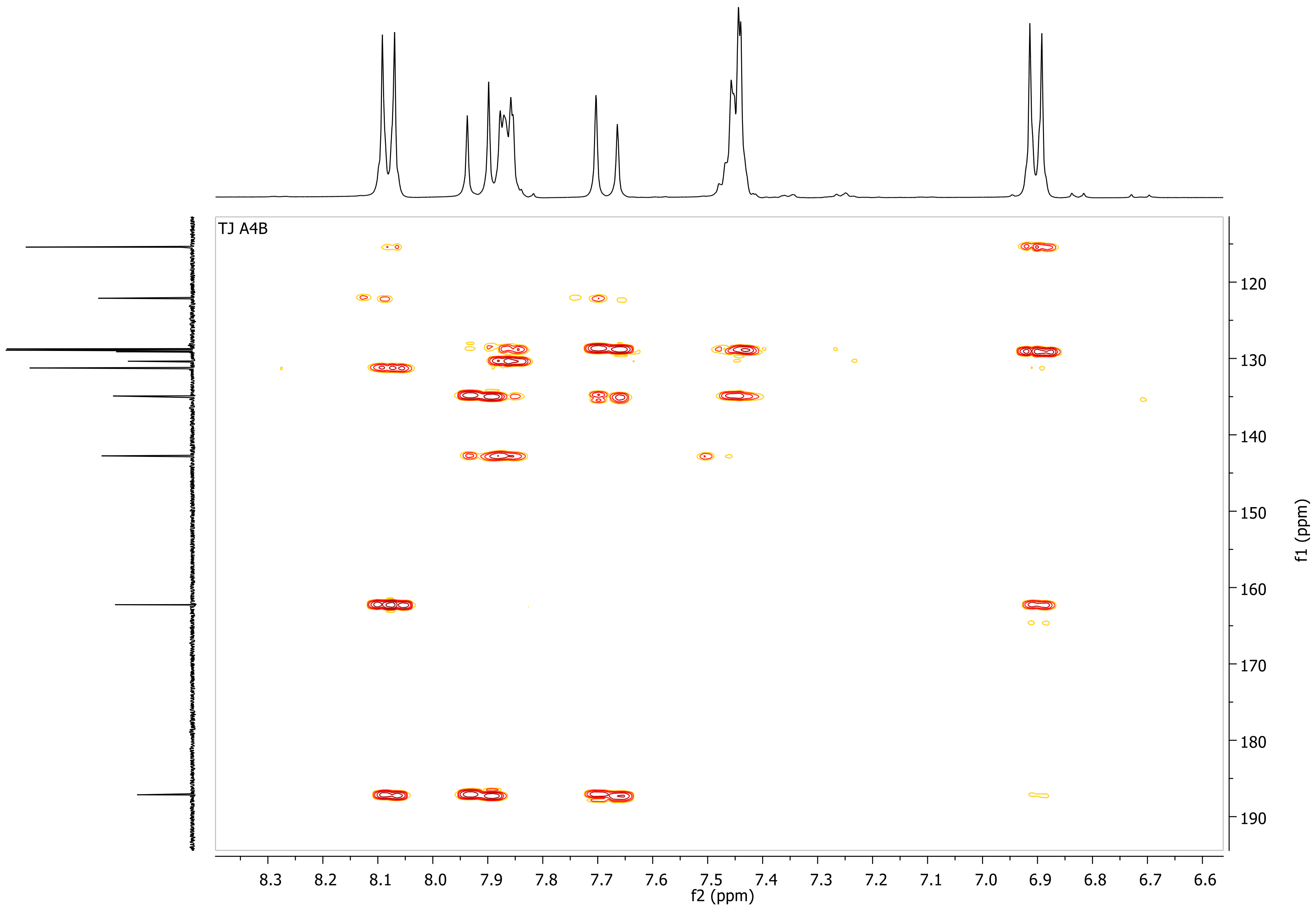

Figure S5: HMBC spectrum of 4'-hydroxychalcone (*trans*-**1**).

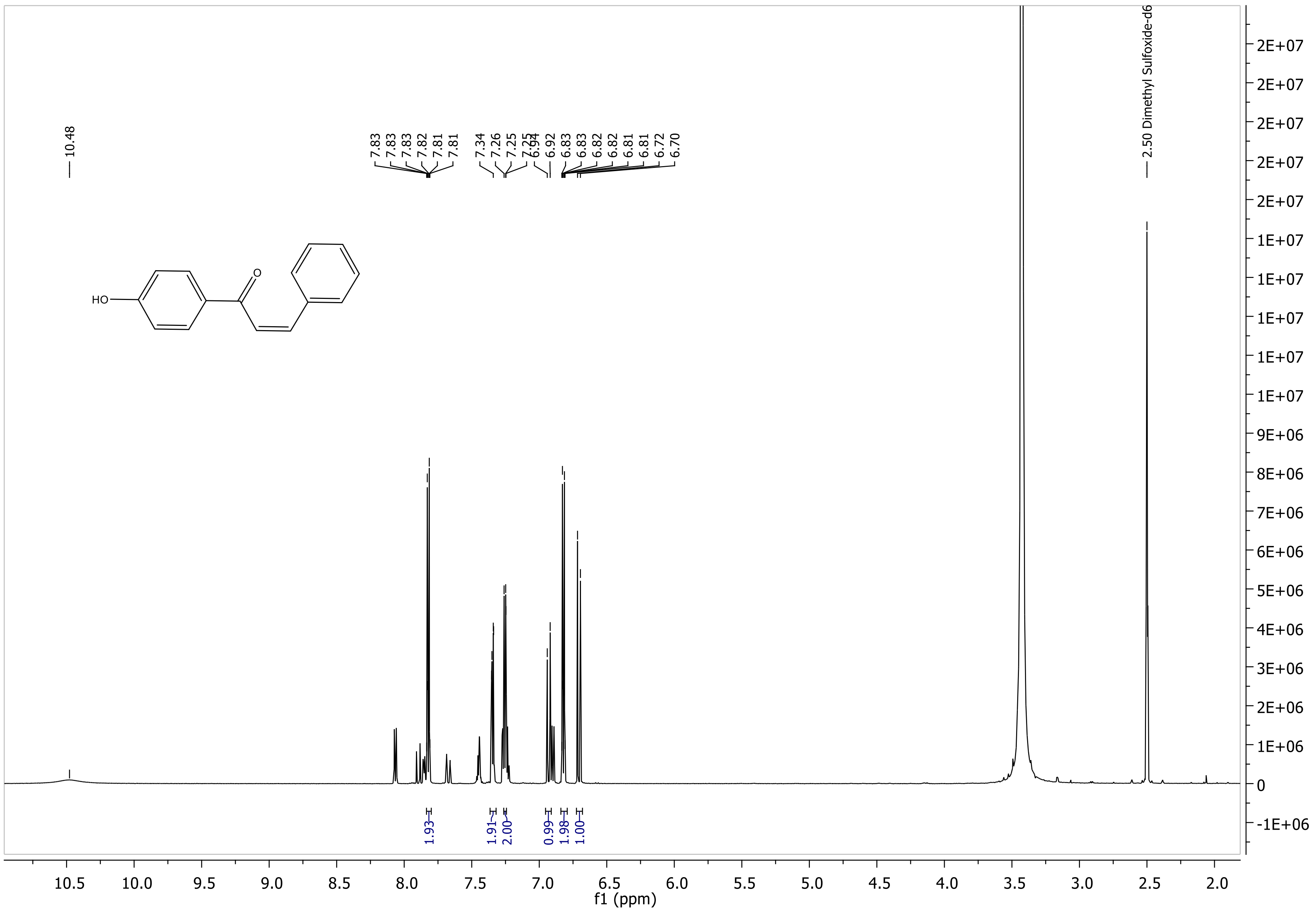

Figure S6: ^{1}H-NMR spectrum of 4'-hydroxychalcone (*cis*-**1**).

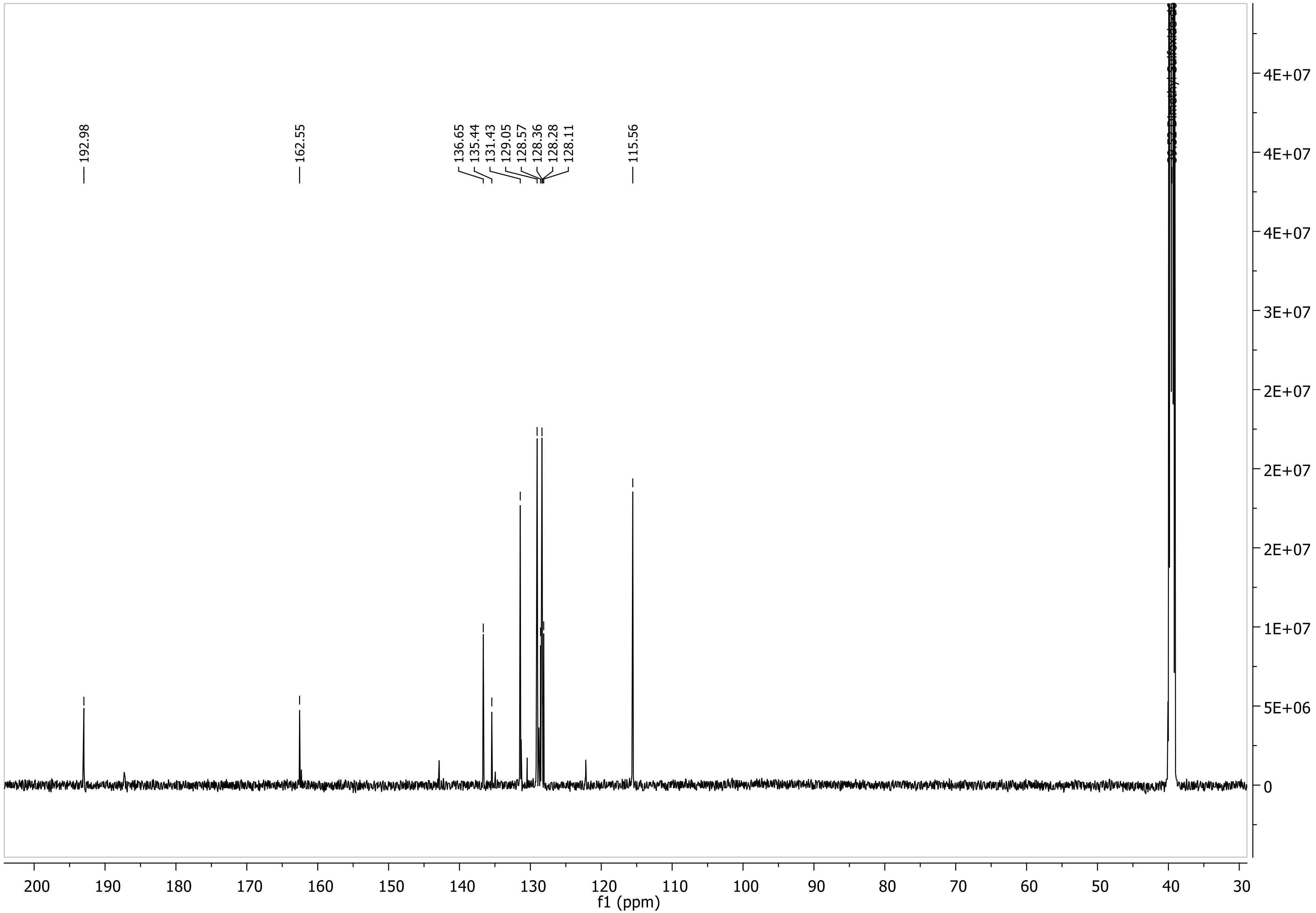

Figure S7: ^{13}C-NMR spectrum of 4'-hydroxychalcone (*cis*-**1**).

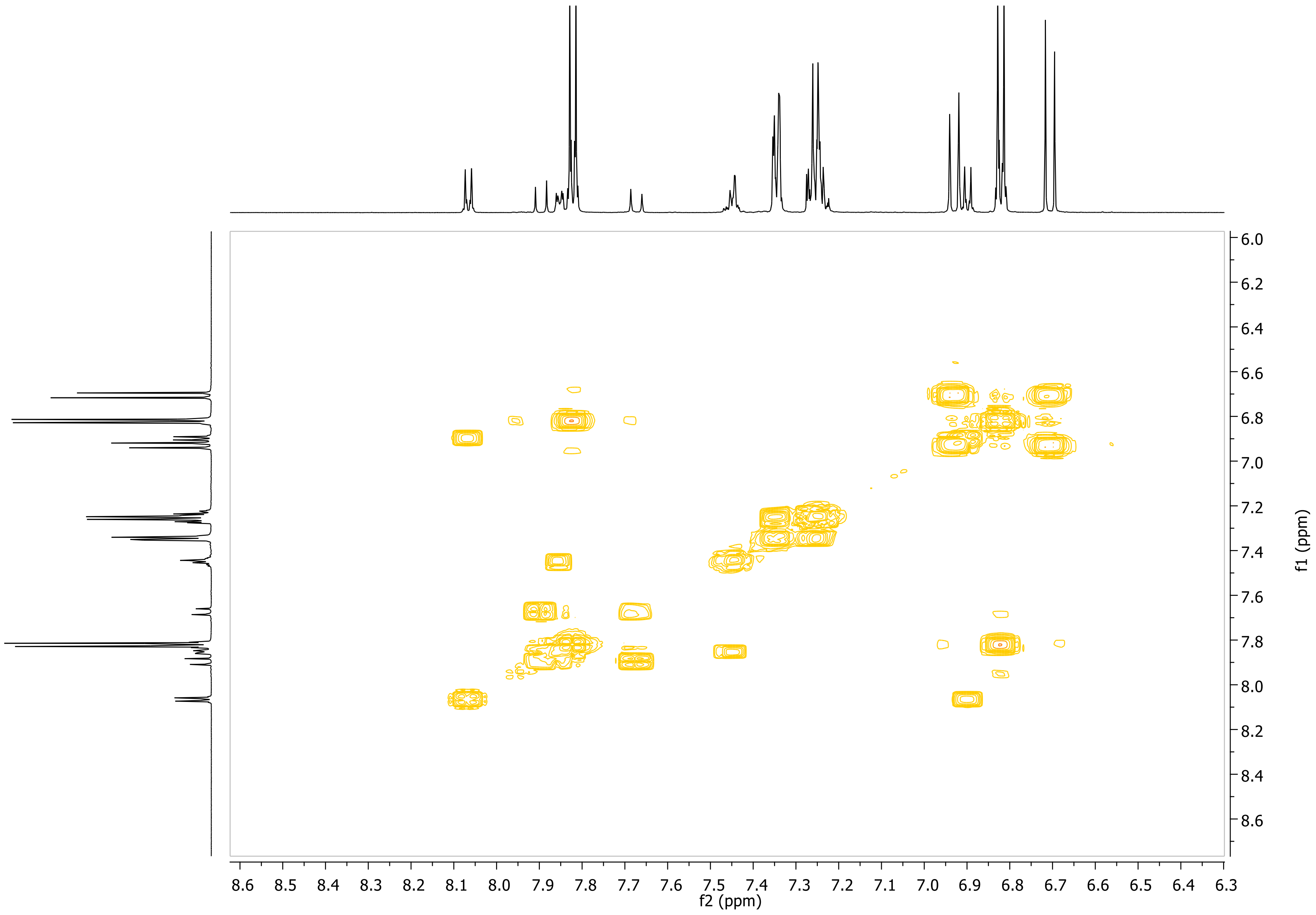

Figure S8: COSY spectrum of 4'-hydroxychalcone (*cis*-**1**).

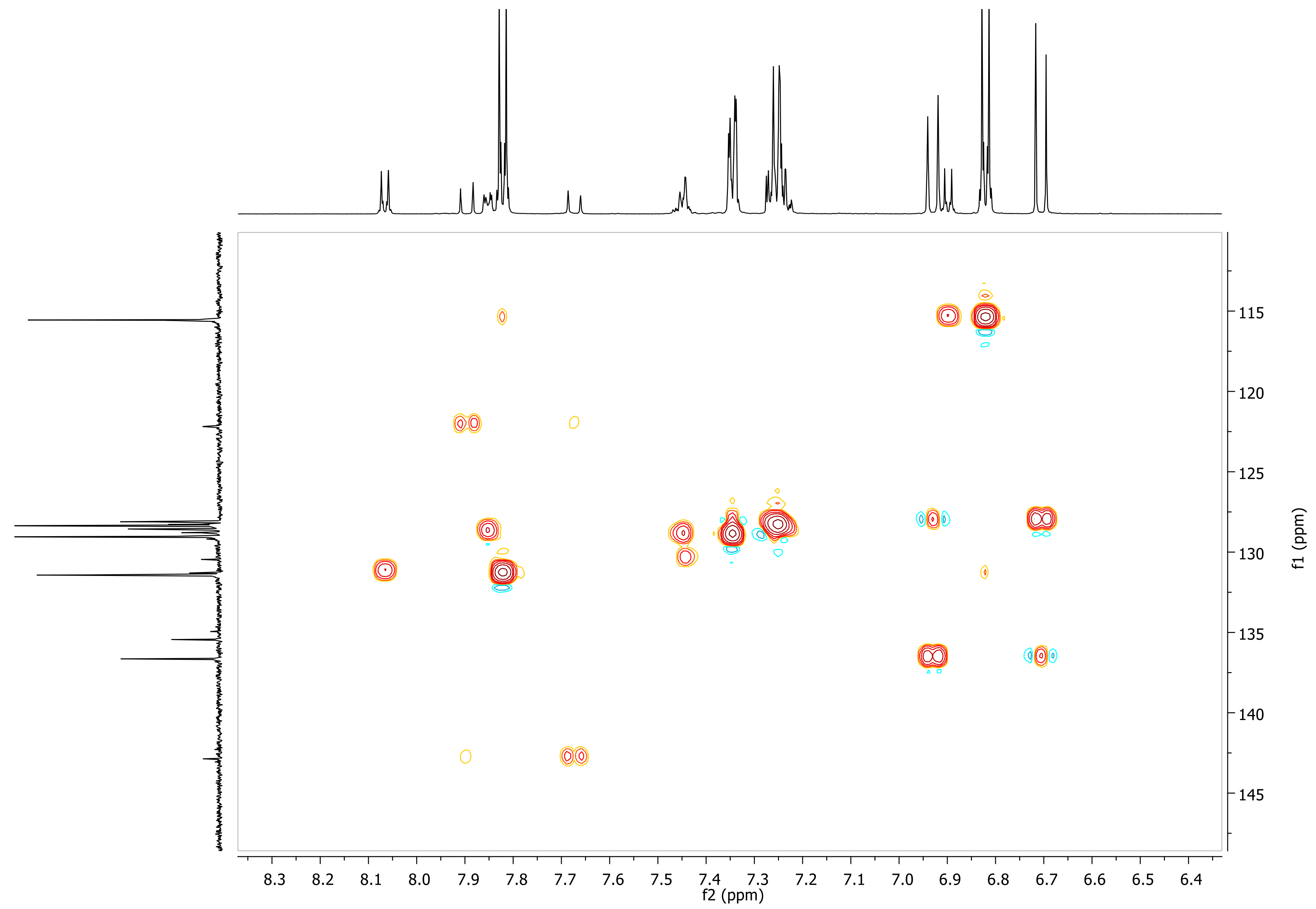

Figure S9: HSQC spectrum of 4'-hydroxychalcone (cis-**1**).

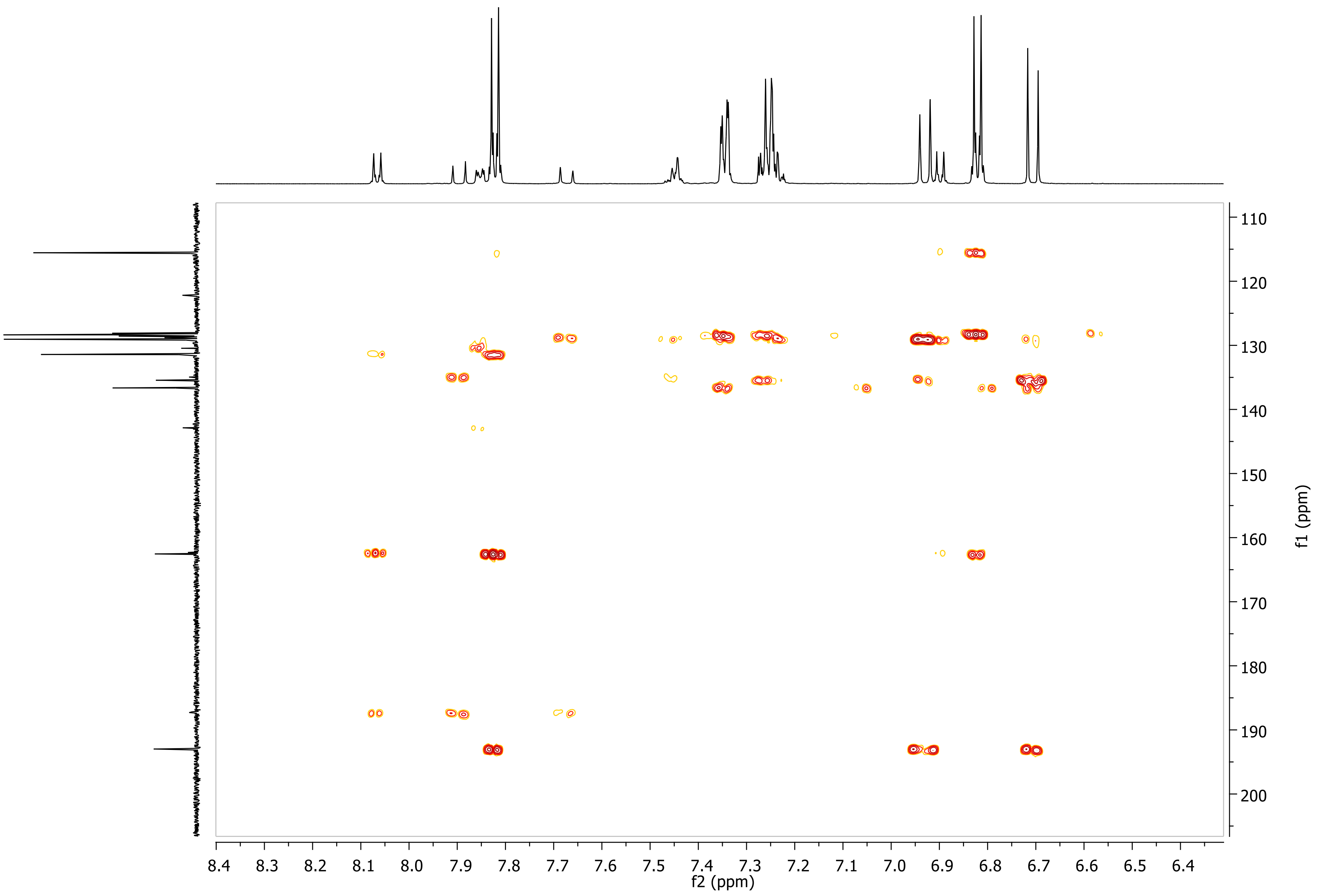

Figure S10: HMBC spectrum of 4'-hydroxychalcone (*cis*-**1**).

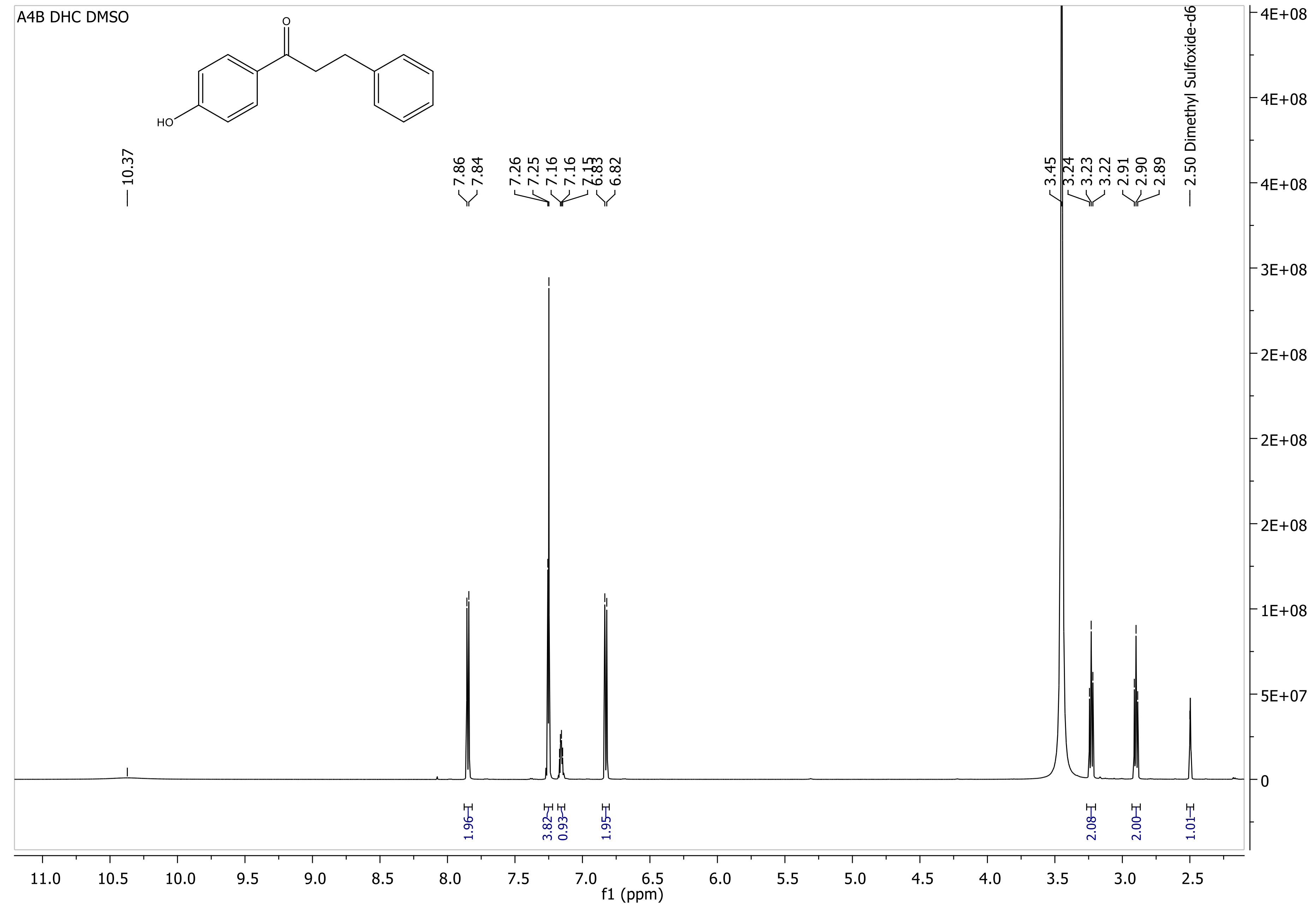

Figure S11: ^{1}H-NMR spectrum of 4’-hydroxy-dihydrochalcone (**2**).

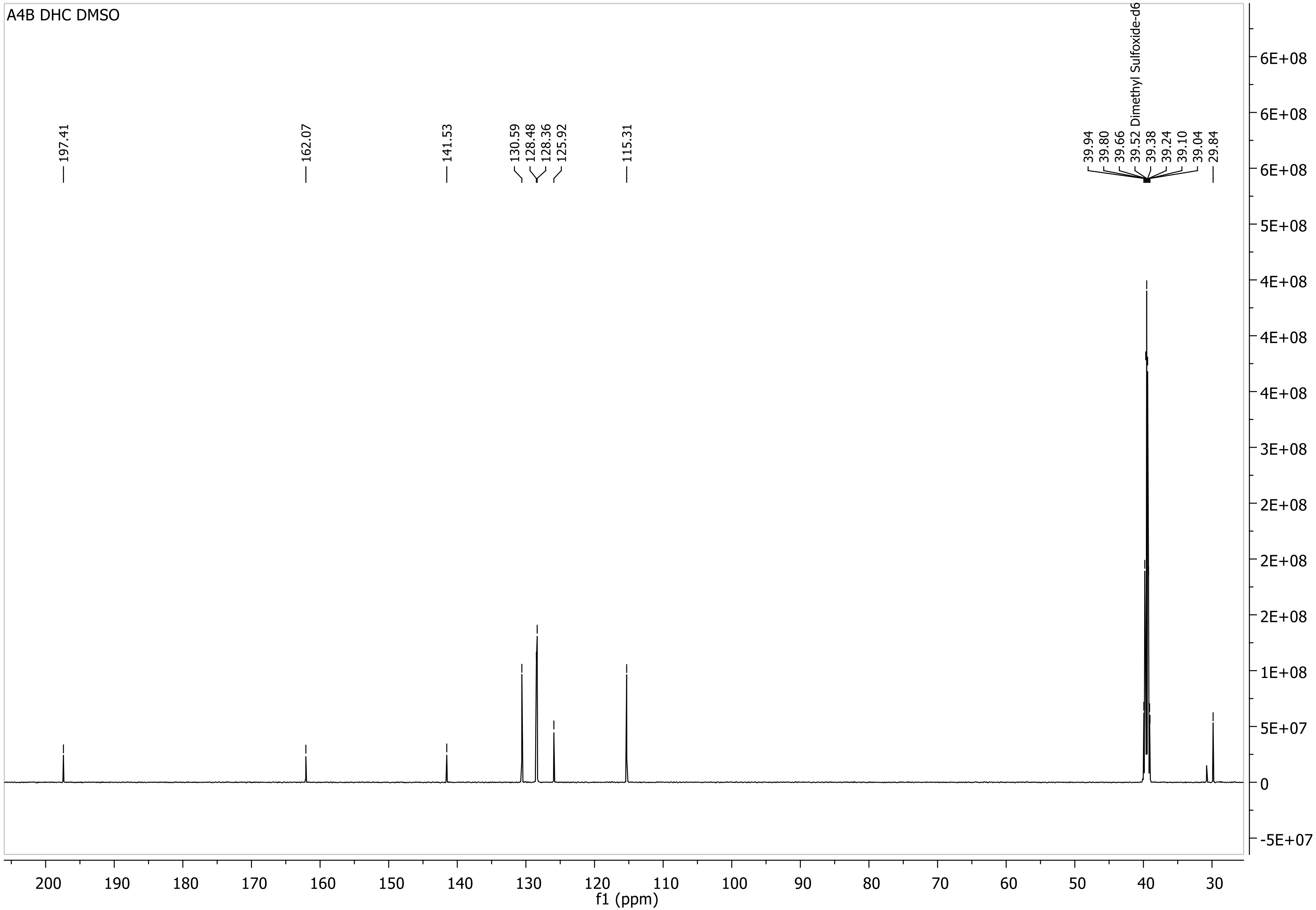

Figure S12: ^{13}C-NMR spectrum of 4’-hydroxy-dihydrochalcone (**2**).

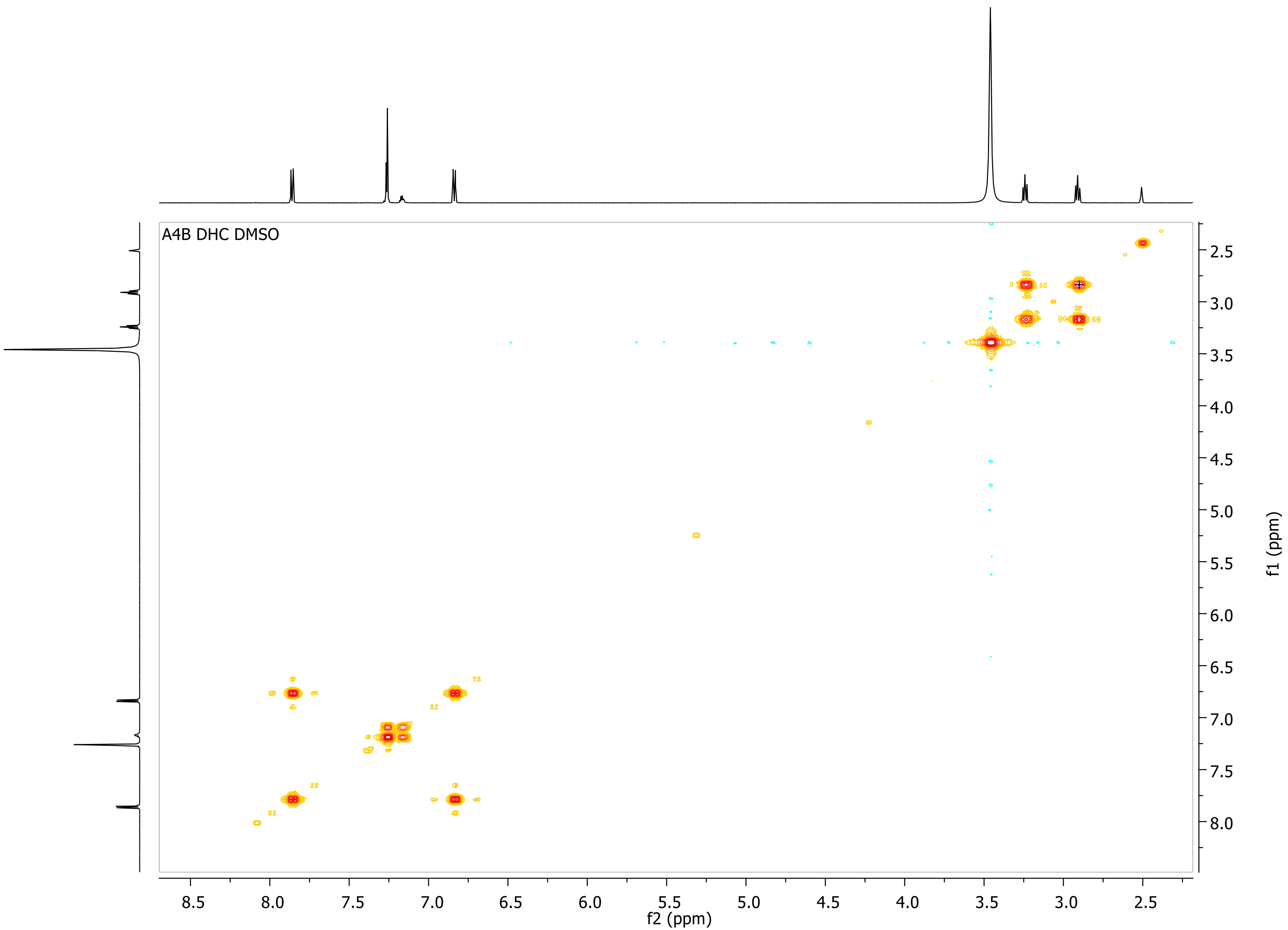

Figure S13: COSY spectrum of 4’-hydroxy-dihydrochalcone (**2**).

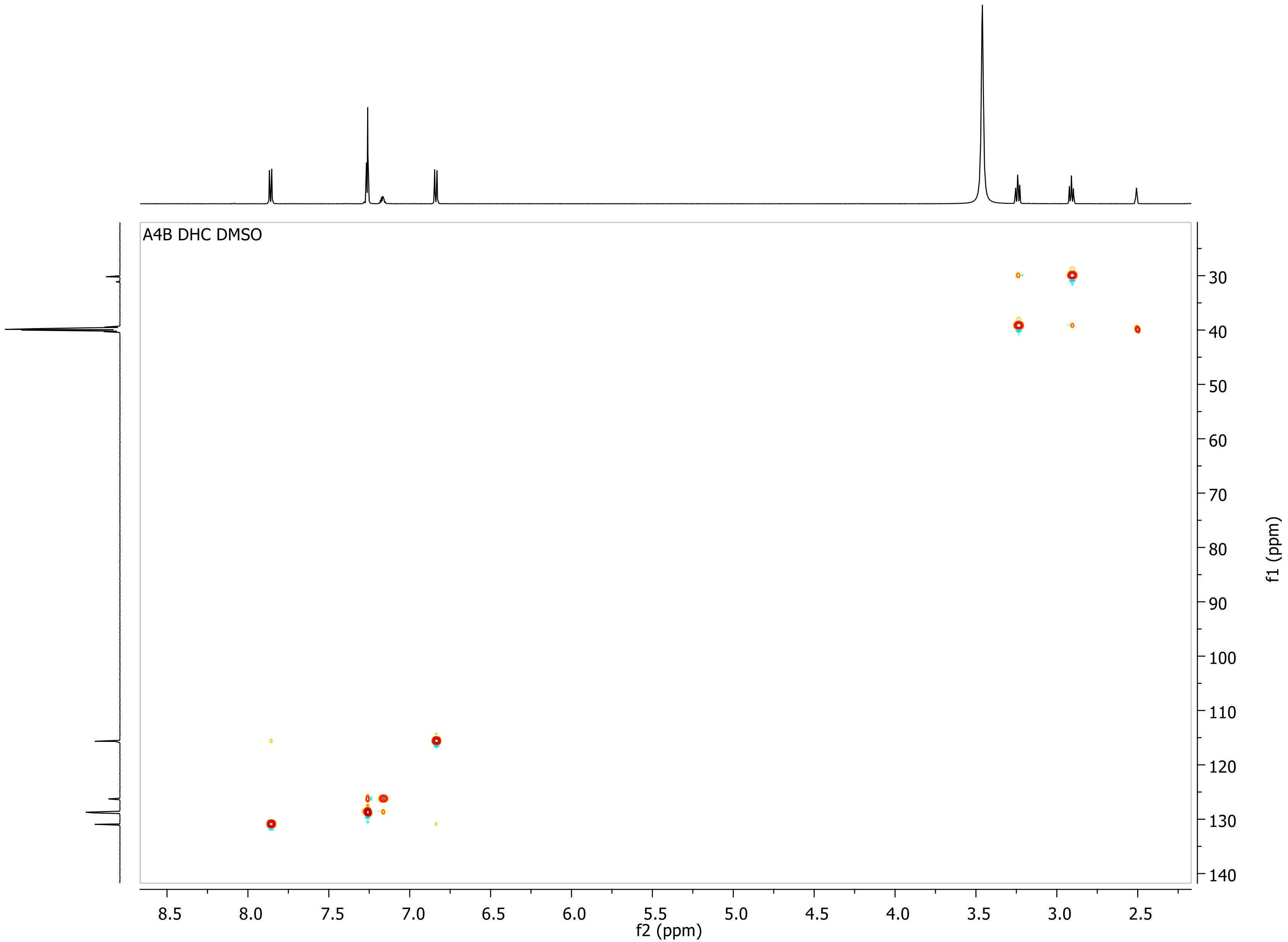

Figure S14: HSQC spectrum of 4'-hydroxy-dihydrochalcone (**2**).

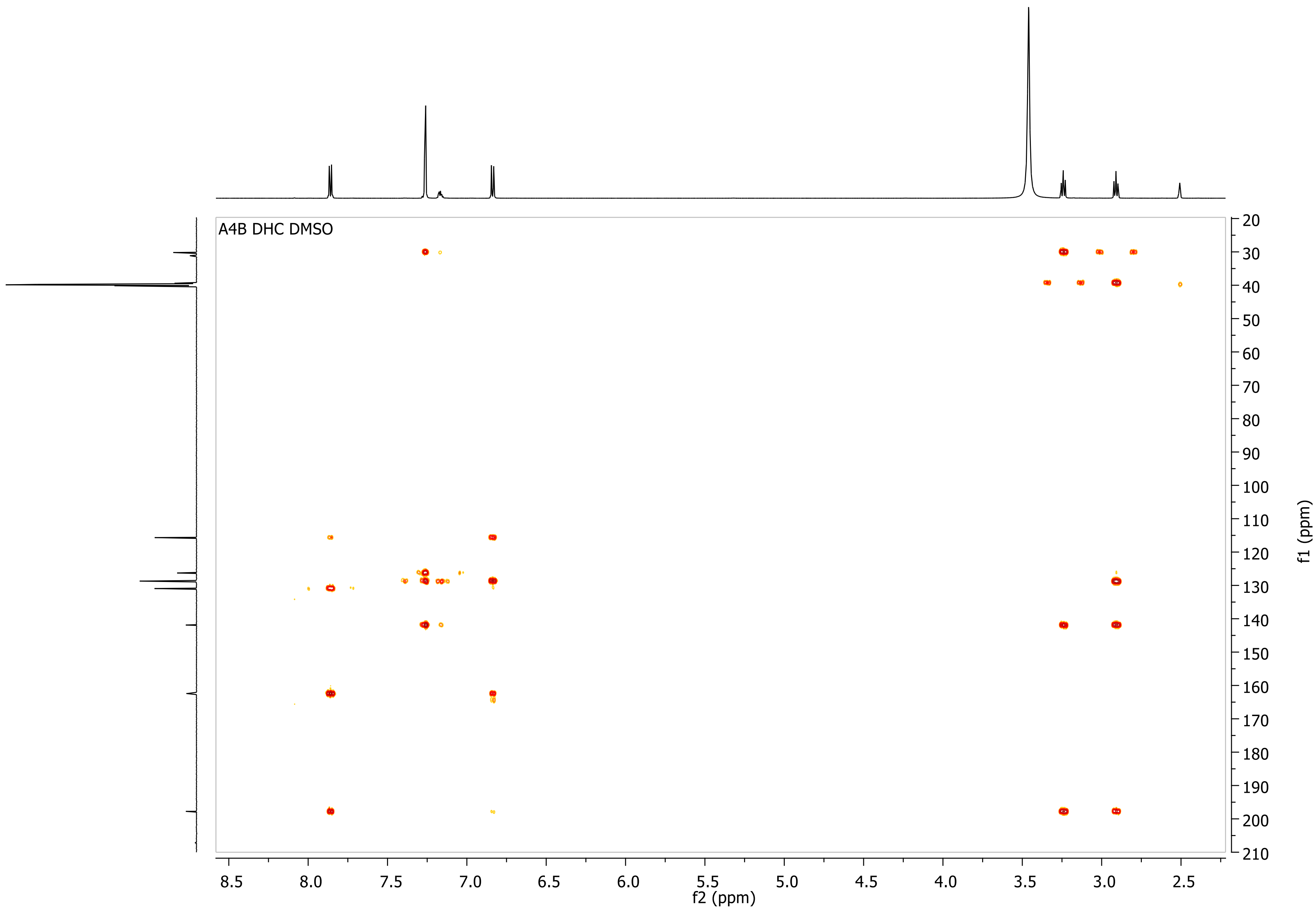

Figure S15: HMBC spectrum of 4’-hydroxy-dihydrochalcone (**2**).
